# *Mycoplasma hyorhinis* infection promotes TNF-α signaling and SMAC mimetic-mediated apoptosis in human prostate cancer

**DOI:** 10.1016/j.heliyon.2023.e20655

**Published:** 2023-10-11

**Authors:** Jin Koo Kim, Insoon Chang, Younghun Jung, Zach Kaplan, Elliott E. Hill, Russell S. Taichman, Paul H. Krebsbach

**Affiliations:** a Division of Oral and Systemic Health Sciences, University of California, Los Angeles School of Dentistry, Los Angeles, CA, USA; b Section of Endodontics, University of California, Los Angeles School of Dentistry, Los Angeles, CA, USA; c Department of Periodontics and Oral Medicine, University of Michigan School of Dentistry, Ann Arbor, MI, USA; d Department of Biologic and Materials Sciences, University of Michigan School of Dentistry, Ann Arbor, MI, USA; e Department of Neurology, Boston University School of Medicine, Boston, MA, USA; f Department of Periodontics, University of Alabama at Birmingham, Birmingham, AL, USA

**Keywords:** *Mycoplasma hyorhinis*, Prostate cancer, TNF-α, NF-κB, SMAC mimetic, AZD5582

## Abstract

Growing evidence suggests an association between Mycoplasma infections and the development and progression of prostate cancer (PCa). In this study, we report that chronic and persistent *M. hyorhinis* infection induced robust TNF-α secretion from PCa cells. TNF-α secreted from *M. hyorhinis*-infected PCa cells subsequently led to activation of the NF-κB pathway. Chronic *M. hyorhinis* infection induced gene expression of pro-inflammatory cytokines and chemokines in a NF-κB-dependent manner and promoted cell proliferation, migration, and invasion in PCa cells. The elimination of *M. hyorhinis* in PCa cells significantly blocked TNF-α secretion, gene expression of cytokines and chemokines, migration, and invasion in PCa cells, suggesting *M. hyorhinis*-induced TNF-α plays an important role to promote malignant transformation of PCa. Furthermore, second mitochondria-derived activator of caspases (SMAC) mimetics potentiated caspase activation and cell death in *M. hyorhinis*-infected PCa by antagonizing inhibitor of apoptosis proteins (IAPs) activity. Tissue microarray analysis indicated that TNF-α is co-expressed in *M. hyorhinis-*infected human patient tissues. Findings from this study advance our understanding of the mycoplasma-oncogenesis process and suggest the potential for new approaches for preventions, diagnosis, and therapeutic approaches against prostate cancers.

## Highlights

• *M. hyorhinis* induces TNF-α secretion from PCa cells.
• *M. hyorhinis* promotes TNF-α expression *in vivo*.
• *M. hyorhinis* infection alters invasion and migration capacity of PCa cells.
• *M. hyorhinis*-induced TNF-α elicits cytokine/chemokine gene expression by NF-κB.
• SMAC mimetic treatment leads to apoptosis of *M. hyorhinis*-infected PCa cells.

## 1 Introduction

Prostate cancer (PCa) is the second most frequently diagnosed cancer in men in the Western world [1]. Despite scientific and technological progression towards unveiling the molecular mechanisms and risk factors involved in PCa, PCa remains the second leading cause of cancer mortality in the U.S. and the fifth leading cause of death in men worldwide [1,2]. Recently, numerous laboratories have suggested that chronic inflammation associated with infectious microbes may contribute to tumor initiation, progression, metastasis, and resistance to chemotherapeutic drugs [[3], [4], [5], [6], [7]]. Thus, identifying molecular links between these microbes and cancer, and understanding the detailed mechanisms underlying their cancer-promoting activity may positively impact the development of novel cancer control strategies.

Mycoplasma is a small, self-replicating mollicute genus of bacteria that lacks a cell wall [8]. Due to the absence of a cell wall, mycoplasma species are pleomorphic which makes them naturally resistant to antibiotics that target cell wall synthesis [4,[9], [10], [11]]. The association between chronic mycoplasma infection and cancer was first reported in the 1960s and suggested that mycoplasma infection caused chromosomal changes [11,12]. Since then, the oncogenic potential of mycoplasma and its contribution to the process of malignant transformation has been studied in several types of cancers [5,10,11]. Growing evidence indicates that chronic infection due to several mycoplasma strains including *Mycoplasma hyorhinis* (*M. hyorhinis*) also promotes malignant transformation of human prostate cells [4,[7], [8], [9],^11^,[13], [14], [15]]. Furthermore, a strong association between mycoplasma species and PCa in biopsies from patients with high-grade prostatic intraepithelial neoplasia or PCa has been demonstrated [2]. For example, it was found that *M. hominis* levels were significantly higher in patients with benign prostatic hyperplasia or PCa, while no mycoplasma species were detected in control samples from lesion-free men [2].

Tumor necrosis factor-alpha (TNF-α) is a potent proinflammatory cytokine that may play an important role in tumorigenesis and progression of PCa [[16], [17], [18], [19], [20], [21], [22], [23], [24], [25]]. Cellular responses to TNF-α are mediated by activation of several pathways including nuclear factor kappa-light-chain-enhancer of activated B cells (NF-κB), protein kinase C-alpha (PKC-α), activator protein 1 (AP-1), or reactive oxygen species (ROS) production [21,22]. TNF-α is produced by cancer cells as well as the tumor microenvironment and can function as both pro-survival and pro-apoptotic factors based on its level of expression [16,18,24]. High expression of TNF-α in the serum of cancer patients and in pre-cancerous and tumor tissues has been associated with tumor proliferation, angiogenesis, invasion and metastases, and resistance to chemotherapeutic agents in several cancers [18,19,22,23,26]. A serial analysis of the serum of PCa patients also showed that significantly elevated levels of TNF-α were correlated with metastatic tumors and the extent of the disease [27].

The cellular inhibitor of apoptosis 1 and 2 (c-IAP1 and c-IAP2) are known to play direct roles in apoptosis regulation in many cancers and also have been reported to regulate TNF-α-mediated NF-κB activation [28]. In the last few decades, extensive research led to the development of small molecules mimicking second mitochondrial-derived activator of caspases (SMAC, also known as DIABLO), which directly bind to IAP proteins and antagonize their activity [29]. In this study, we report that persistent mycoplasma infection promotes the secretion of a significantly high level of TNF-α in PCa cells leading to activation of the NF-κB pathway and *in vitro* characteristics of PCa progression. In addition, our data demonstrate that SMAC mimetics significantly induce cell death in *M. hyorhinis*-infected PCa cells exhibiting aggressive migration and invasive behavior.

## 2 Materials and methods

### 2.1 Chemicals

SMAC mimetics, AZD5582 (Catalog number: S7362), Birinapant (S7015), LCL161 (S7009), SM-164 (S7089), ASTX660 (S8681), and AT406 (SM-406) (S2754) were purchased from Selleck Chemicals (Houston, TX). IKK-2 inhibitor VI (401483) was purchased from Calbiochem (Sigma-Aldrich, St. Louis, MO). All compounds were diluted in DMSO according to manufacturer's recommendations.

### 2.2 Cell culture

Human primary fibroblasts (HF) [30], human primary dental pulp cells (DPC) [31], and human cancer cell lines (PC3, C4–2B, DU145, LNCaP, MDA-MB-231, MCF-7, MCF10A, HeLa, MG-63; American Type Culture Collection (ATCC), Manassas, VA) were grown in Dulbecco's modified Eagle's medium (DMEM, Invitrogen, Carsbad, CA) supplemented with 10 % FBS and 1 % penicillin/streptomycin. Human dermal microvascular endothelial cells (HDMEC; Cambrex, Walkersville, MD) were cultured in endothelial growth medium-2 for microvascular cells (EGM2-MV; Cambrex). Mycoplasma-free PCa cells from ATCC were seeded into T-75 flasks and infected with *Mycoplasma hyorhinis* (3 × 10^7^ colony-forming units (CFU), Catalog # 17981, ATCC) at a multiplicity of infection (MOI) of 5 for 2 passages and then passaged twice a week for 6 weeks without further infection. Four weeks after infection, *M. hyorhinis* was eliminated in cell cultures for 3 weeks by Mycoplasma-EX kit (PK-CC91-4003, PromoCell GmbH, Heidelberg, Germany) following the manufacturer's instructions. The chronic infection or elimination of *M. hyorhinis* in PCa cells was confirmed by Western blotting using monoclonal anti-*M. hyorhinis* (P70 surface antigen) antibody that does not cross-react with other species of Mycoplasma (Kerafast company confirmed by experiments).

### 2.3 Enzyme-linked immunosorbent assay

Supernatants of cell cultures were collected and centrifuged to eliminate cell debris. TNF-α expression was determined by a human TNF-α Quantikine ELISA kit (DTA00C, R&D systems, Minneaplis, MN) following the manufacturer's instructions. The fluorescence was quantified via a TECAN microplate reader (TECAN US, Durham, NC). Data were normalized to cell number in each test.

### 2.4 Quantitative reverse transcription PCR (qRT-PCR)

Total RNA was extracted from cells using TRIzol reagent (15596026, Invitrogen) and converted into cDNA using a SuperScript First-Strand Synthesis System (11904018, Invitrogen) following the manufacturer's instructions. qRT-PCR was performed using a SYBR Green supermix (1708880, Bio-Rad). The PCR product was detected as an increase in fluorescence with total 46 cycle numbers (Ct) in a CFX96 Real Time PCR System (Bio-Rad). GAPDH was used as an internal control for the normalization of target gene expression. Primer sequences are listed in Table S3. RNA quantity (CR) was normalized to the housekeeping gene GAPDH by using the formula CR = 2^(46−Ct of sample)−(46−Ct of control)^. The threshold cycle (Ct) is the cycle at which a significant increase in fluorescence occurs.

### 2.5 Western blot analysis

Whole cell lysates were prepared from cells, separated on Novex 4–20 % gradient tris-glycine gel (XV04205PK20, Invitrogen) and transferred to PVDF membrane. The membranes were incubated with 5 % milk for 1 h and incubated with primary antibodies overnight at 4 °C. Primary antibodies used were as follows: *M. hyorhinis* (P70 surface antigen) (1:1000, EMZ104, Kerafast, Boston, MA), IκB-α (1:1000, 9242, Cell Signaling Technology (CST), Danvers, MA), TNF-α (1:1000, 8184, CST), Caspase-8 (1:1000, 9746, CST), Cleaved Caspase-8 (1:1000, 9496, CST), Caspase-9 (1:1000, 9502, CST), Cleaved Caspase-3 (1:1000, 9661, CST), poly (ADP-ribose) polymerase (PARP) (1:1000, 9542, CST), c-IAP1 (1:1000, 7065, CST), c-IAP2 (1:1000, 3130, CST), β-actin (1:5000, 4970, CST), and α-tubulin (1:5000, sc-8035, Santa Cruz Biotechnology, Santa Cruz, CA). Blots were incubated with peroxidase-coupled secondary antibodies (Promega, Madison, WI) for 1 h, and protein expression was detected with SuperSignal West Pico Chemiluminescent Substrate (Thermo Scientific, Rockford, IL).

### 2.6 Immunoprecipitation

Supernatants of *M. hyorhinis*-infected cell cultures were collected and centrifuged to eliminate cell debris. Half of the supernatants (3 mL) were incubated with 15 μg of monoclonal anti-human TNF-α antibody (MAB610, R&D systems, Minneapolis, MN) for 2 h at 4 °C and then incubated with 120 μl of Protein A/G PLUS-Agarose (sc-2003, Santa Cruz) for 1 h at 4 °C. Immunoprecipitates were analyzed for TNF-α by Western blot analysis. Cleared supernatants were used for PC3 cell treatment.

### 2.7 Immunofluorescence staining

PCa tissue microarrays (TMA, BC19021a, US Biomax, Rockville, MD) were deparaffined and the antigens were retrieved using a pressure cooker. The tissues were washed three times in PBS, permeabilized with PBS containing 0.1 % Triton X-100 for 10 min, and then incubated with 2 % BSA for 1 h. The tissues were incubated overnight at 4 °C with mouse anti-*M. hyorhinis* (P70 surface antigen) (1:500, EMZ104, Kerafast, Boston, MA) and rabbit anti-TNF-α (1:400, 8184, Cell Signaling). After washing three times in PBS, cells were incubated with Alexa Fluor 594 or 488 coupled secondary antibodies for 1 h, washed three times in PBS, and then mounted using Vectashield antifade mounting medium with DAPI (H-1200, Vector Laboratories, Burlingame, CA). Images were taken by whole slide scanning from UCLA Translational Pathology Core Laboratory and using Aperio ImageScope software (Leica Biosystems).

### 2.8 Cell proliferation and viability assay

For cell proliferation assays, PCa cells (1 × 10^5^) were seeded onto 100-mm culture dishes for 2- and 4-day analysis. Cell counting was performed using trypan blue. For cell viability assays, PCa cells (1 × 10^5^) were seeded onto 12-well culture plates. After 24 h, cells were treated with IKK2 inhibitor VI or SMAC mimetics at 37 °C for 48 h. Cell death was determined by trypan blue assay. Apoptosis was confirmed by Western blotting using the antibodies of Caspase-8, cleaved Caspase-8, Caspase-9, cleaved Caspase-3, and PARP.

### 2.9 Scratch wound healing assays

PCa cells (3 × 10^6^) were seeded into 60-mm culture dishes. After 24 h, cells were scratched with a P200 pipette tip and washed with DMEM medium containing 10 % FBS. After 15 h, images representative of wound healing were captured with a Nikon Eclipse TE2000-S microscope.

### 2.10 Cell invasion assay

PCa cells (5 × 10^5^) were seeded onto Corning Matrigel Invasion Chamber 24-Well Plate 8.0 μm (354480, Corning, Bedford, MA), with 1 mL of DMEM medium without FBS within the top chamber, and 1 mL of DMEM medium with 10 % FBS on the bottom of the plate. After 24 h, invasive cells adhered to the bottom chamber were stained with Hema 3™ Stat Pack (123–869, Fisher, Pittsburgh, PA) according to the manufacturer's instructions. Stained cells were counted and images were taken with a Nikon Eclipse TE2000-S microscope.

### 2.11 Statistical analysis

Results are presented as mean ± standard deviation (SD). Significance of the difference between two measurements was determined by unpaired the Student's *t*-test, and multiple comparisons were evaluated by the Newman-Keuls multiple comparison test. Values of *p* < 0.05 were considered significant. Regression analysis between *M. hyorhinis* (P70) and TNF-α staining intensity were conducted using GraphPad Prism version 7 software.

## 3 Results

### 3.1 *M. hyorhinis* induces TNF-α secretion from PCa cells

**Fig. 1:**
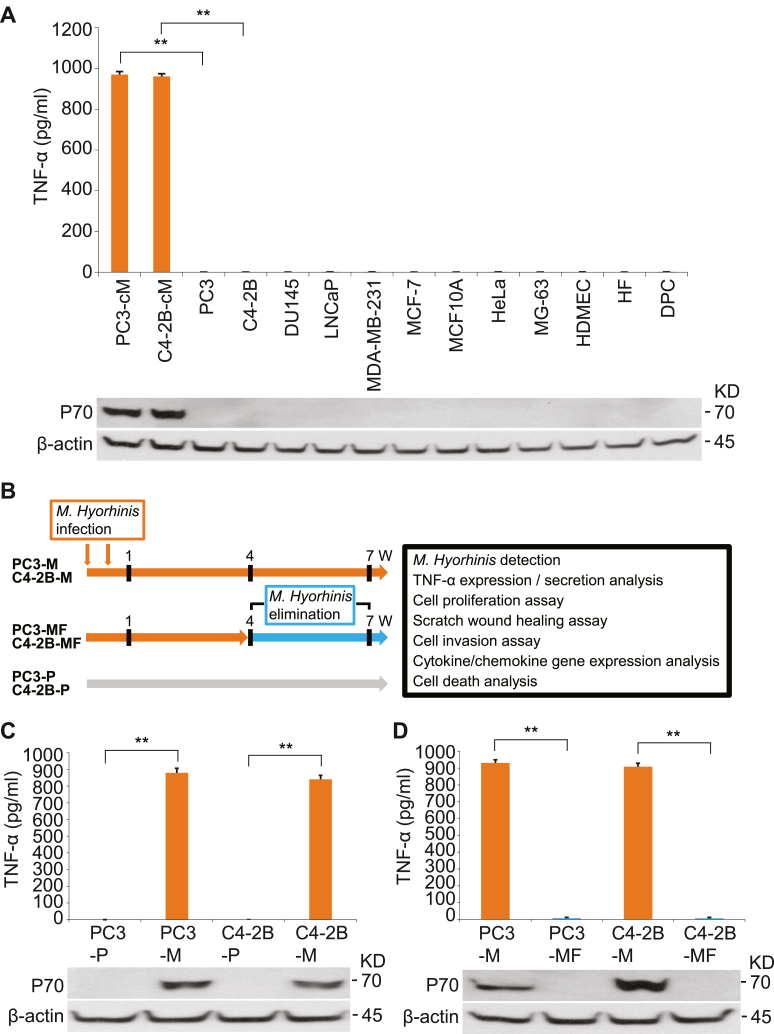
M. hyorhinis promotes TNF-α secretion from PCa cells. (A), relative TNF-α secretion levels in M. hyorhinis-contaminated PCa cells (PC3-cM and C4–2B-cM) and mycoplasma-free cells; multiple cancer cells (PC3, C4–2B, DU145, LNCaP, MDA-MB-231, MCF-7, MCF10A, HeLa, MG-63), HDMEC, HF, and DPC via ELISA. (B), experimental design. The parental PCa cells (PC3–P and C4–2B–P) were infected with M. hyorhinis (3 × 107 CFU, 5 MOI) for 2 passages and then passaged twice a week for 6 weeks without further infection (PC3-M and C4–2B-M). For PC3-MF and C4–2B-MF cells, 4 weeks after infection, M. hyorhinis was eliminated in cell cultures for 3 weeks. Seven weeks after infection, *in vitro* assays and analyses were performed using these PCa cells. (C), relative TNF-α secretion levels in the parental (PC3–P and C4–2B–P) and M. hyorhinis-infected (PC3-M and C4–2B-M) PCa cells. (D), relative TNF-α secretion levels in PC3-M and C4–2B-M and previously infected PCa cells after elimination of M. hyorhinis (PC3-MF and C4–2B-MF). M. hyrorhinis infection was confirmed by Western blotting using specific anti-M. hyorhinis (P70 surface antigen) antibody. βactin was used as a loading control. See full images in Supplementary Figure S4. All results represent mean ± SD values from triplicate assays, and the experiments were repeated three times. **p < 0.0001. HDMEC, human dermal microvascular endothelial cells; HF, human primary fibroblasts; DPC, human primary dental pulp cells; CFU, colony-forming units; MOI, multiplicity of infection.

The pro-inflammatory cytokine TNF-α was detected in the supernatants collected from mycoplasma-contaminated PCa cells (PC3-cM and C4–2B-cM) and mycoplasma-free cells; multiple human cancer cells (PC3, C4–2B, DU145, LNCaP, MDA-MB-231, MCF-7, HeLa, MG-63), a non-tumorigenic epithelial cells (MCF10A), human dermal microvascular endothelial cells (HDMEC), human primary fibroblasts (HF), and human primary dental pulp cells (DPC) (Fig. 1A). *M. hyorhnis* is one of the most prevalent mycoplasma contaminants of tissue culture used in research and commercial production. *M. hyorhinis* infection was confirmed with Western blotting analysis using specific anti-*M. hyorhinis* (P70 surface antigen) antibody that does not cross-react with other species of mycoplasma (Kerafast). While levels of TNF-α were not observed in *M. hyorhinis*-free cell lines, PC3-cM and C4–2B-cM cells secreted significantly high levels of TNF-α (Fig. 1A). To rule out the possibility that TNF-α was induced by a mycoplasma other than *M. hyorhinis*, mycoplasma-free PCa cells (PC3–P and C4–2B–P cells) were treated with *M. hyorhinis* for 7 weeks (PC3-M and C4–2B-M cells, Fig. 1B). We found that long-term infection of *M. hyorhinis* induced the secretion of TNF-α (Fig. 1C). In addition, PC3-M and C4–2B-M cells expressed significantly high levels of *TNF* mRNA and TNF-α protein compared to their parental cells (PC3–P and C4–2B–P) and the triple-negative breast cancer cell line, MDA-MB-231 that is known to make TNF-α (Fig. S1). Interestingly, when *M. hyorhinis* microbes were eliminated from PC3-M and C4–2B-M cells (PC3-MF and C4–2B-MF cells, Fig. 1B), TNF-α levels were attenuated, suggesting that chronic *M. hyorhinis*-exposure has a direct effect on TNF-α expression level of PCa cells (Fig. 1D).

### 3.2 *M. hyorhinis* promotes TNF-α expression *in vivo* and may correlate with cancer progression

**Fig. 2:**
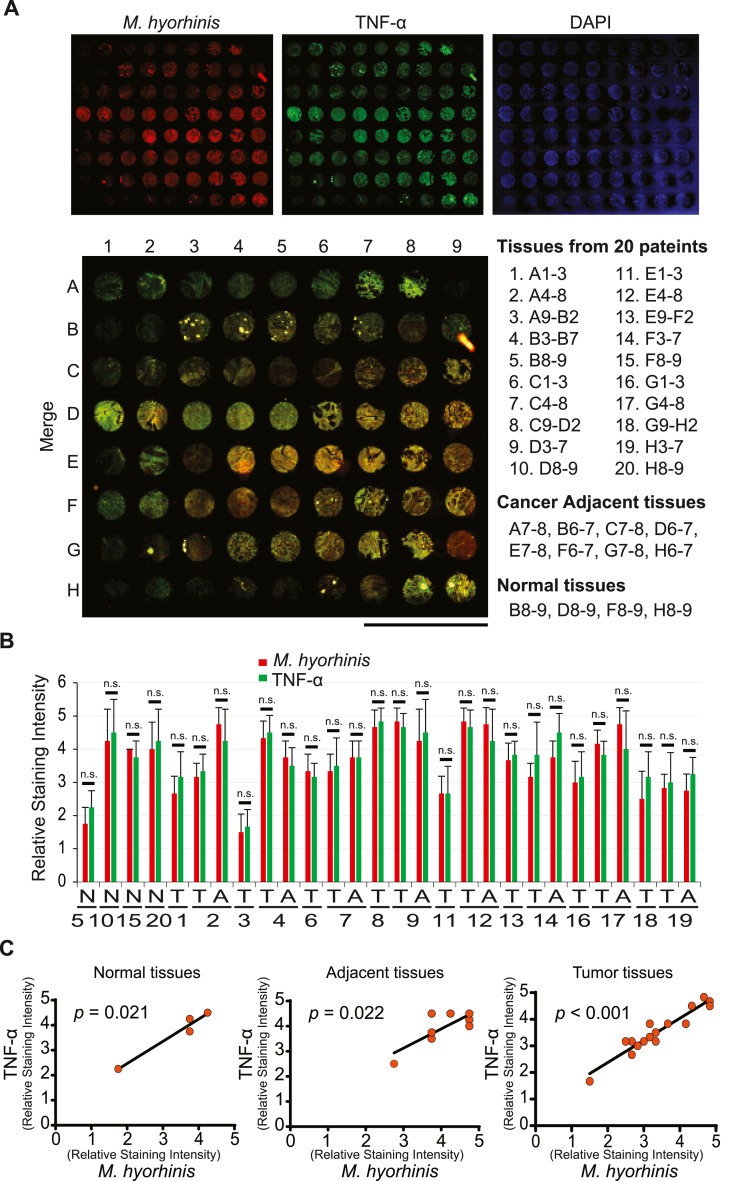
M. hyorhinis infection correlates to TNF-α expression *in vivo*. (A), whole slide scanning images of PCa tissue microarrays. The tissues were analyzed by immunofluorescence staining with M. hyorhinis (P70 surface antigen) and TNF-α antibodies. Scale bars, 7 mm. (B), relative staining intensity of PCa tissue microarray for Normal tissues (N; duplicate cores per case; 4 patients; total n = 8), Adjacent tissues (A; duplicate cores per case; 8 patients; total n = 16), and Tumor tissues (T; triplicate cores per case; 16 patients; total n = 48). Staining intensity was scored blindly by two individuals as absent (1), weak (2), moderate (3), strong (4), and very strong (5). Values are means ± S.D. n.s., not significant. (C), correlation coefficient between M. hyorhinis and TNF-α staining intensity in Fig. 2B represents in normal tissues (r = 0.958), adjacent tissues (r = 0.608), and tumor tissues (r = 0.904), respectively.

Immunofluorescent staining of PCa tissue microarrays (TMA) was performed to determine whether there is a correlation between *M. hyorhinis* infection and TNF-α expression using 72 tissue samples from 20 patients on TMAs (16 cases of adenocarcinoma, 8 cases of adjacent tissue, 4 cases of normal tissue, duplicate or triplicate cores per case) (Fig. 2A). P70 and TNF-α co-stained images revealed that *M. hyorhinis* infection is robust in PCa tissues expressing TNF-α (Fig. 2A). The relative staining intensity quantification also demonstrated that P70 expression approximated TNF-α expression levels in all types of PCa tissue samples, including normal, tumor, and adjacent tissues (Fig. 2B and C). Moreover, higher P70 levels in PCa tissues correlated with a heightened level of TNF-α (Fig. 2B and C), indicating that *M. hyorhinis* infection dose impacts the expression level of TNF-α in PCa. In particular, the mRNA expression profile of Prostate Adenocarcinoma mycoplasma microbiome data (n = 492) from The Cancer Genome Atlas (TCGA, PanCancer Atlas) using cBioPortal analysis (www.cbioportal.org) demonstrated that *TNF* mRNA is highly expressed in 4 % (altered group; n = 20; Log2 Ratio = 3.18; *p* = 1.67E-18) of Prostate Adenocarcinoma patient cases (Table S1 and Fig. S2A). The compelling comparison with the mycoplasma microbiome signature to the altered group showed a log ratio of 0.17 with a *p*-value of 0.0334, revealing that mycoplasma infection is significantly more abundant in a high *TNF* mRNA expressing patient group (Table S1). Although there was the relatively low correlation between *TNF* mRNA expression and mycoplasma infection in TCGA data (Table S1) compared to TMA staining data (Fig. 2), *M. hyorhinis* may have a considerable effect for *TNF* expression in PCa. Furthermore, this percentage of high *TNF* mRNA expressing Prostate Adenocarcinoma patients (altered group; Log2 Ratio = 3.18; *p* = 1.67E-18; Fig. S2A) also presented a greater level of lymph node metastasis than the unaltered group (Fig. S2B). The altered group was also associated with a higher degree of advanced stages of cancer (Fig. S2C). Interestingly, similar mycoplasma microbiome signatures and clinical data were also observed in the high *TNF* mRNA group in patients with different types of tumors, such as uterine corpus endometrial carcinoma and bladder urothelial carcinoma (Table S1 and Fig. S3). Taken together, these data suggest a strong correlation between mycoplasma infection and high TNF-α expressing PCa *in vivo* and altered TNF-α expressing PCa attributes of aggressive tumor malignancy phenotypes.

### 3.3 *M. hyorhinis* infection alters invasion and migration capacity of PCa cells

**Fig. 3:**
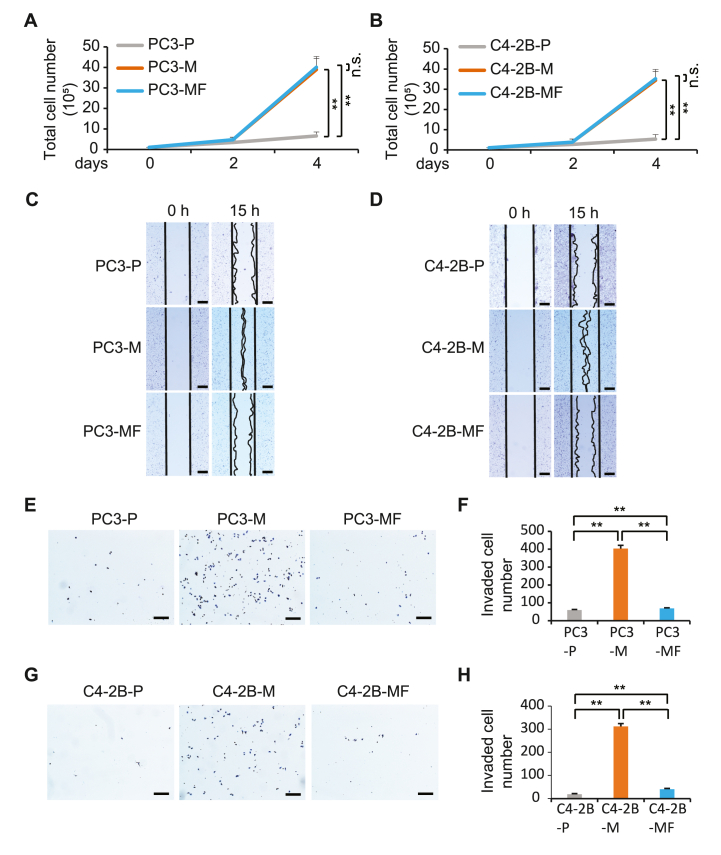
M. hyorhinis infection promotes aggressive transformation of PCa cells. (A and B), cell proliferation assays. PCa cells (1 × 105) were seeded onto 100-mm culture dishes and counted at day 2 and 4. Cell counting was performed using trypan blue. (C and D), scratch wound healing assay. PCa cells (3 × 106) were seeded into 60-mm culture dishes and after 24 h, cells were scratched and after 15 h, images representative of wound healing were captured. Scale bars, 60 μm. E-H, cell invasion assay. PCa cells (5 × 105) were seeded onto Matrigel Invasion Chamber 24-Well Plate. After 24 h, stained invasive cells were counted (F and H) and images were taken (E and G). Scale bars, 100 μm. All results represent mean ± SD values from triplicate assays, and the experiments were repeated three times. **P < 0.0001, n.s., not significant. (For interpretation of the references to colour in this figure legend, the reader is referred to the Web version of this article.)

Because *M. hyorhinis*-induced TNF-α expression in PCa may promote advancement of tumor progression *in vivo*, we assessed the extent to which *M. hyorhinis* infection affected proliferation, invasion, and migration of PCa cells. Cell proliferation assays demonstrated that *M. hyohinis* infection significantly increased proliferation of PC3-M cells compared to the parental PC3 cells. Surprisingly, *M. hyorhinis*-free PC3 cells (PC3-MF), which were once infected with *M. hyorhinis*, maintained a high proliferation rate even after decontamination (Fig. 3A). A similar phenomenon was observed in C4–2B cells (Fig. 3B), suggesting that *M. hyorhinis* infection may cause an irreversible change in the proliferation capacity of PCa cells. The migration assays revealed an increase in cell mobility in PC3-M cells and C4–2B-M cells compared to the parental PC3 and C4–2B cells, respectively (Fig. 3C and D). However, when *M. hyorhinis* was removed in PC3-MF and CB-2B-MF cells, the migration rate slowed and returned to rates comparable to the parental cells (Fig. 3C and D). In results similar to migration assays, invasion assays demonstrated increased invasive properties, a critical step in tumor metastasis, in *M. hyorhinis*-infected PCa cells compared to their parental PC3 and C4–2B cells and this enhanced invasive ability of PC3-M and C4–2B-M cells was reversed in PC3-MF and C4–2B-MF cells (Fig. 3E–H). Together, our data demonstrate that *M. hyorhinis* infection in PCa cells initiates many of the phenotypic characteristics consistent with aggressive tumor progression.

### 3.4 *M. hyorhinis*-induced TNF-α elicits cytokine/chemokine gene expression through NF-κB activation

**Fig. 4:**
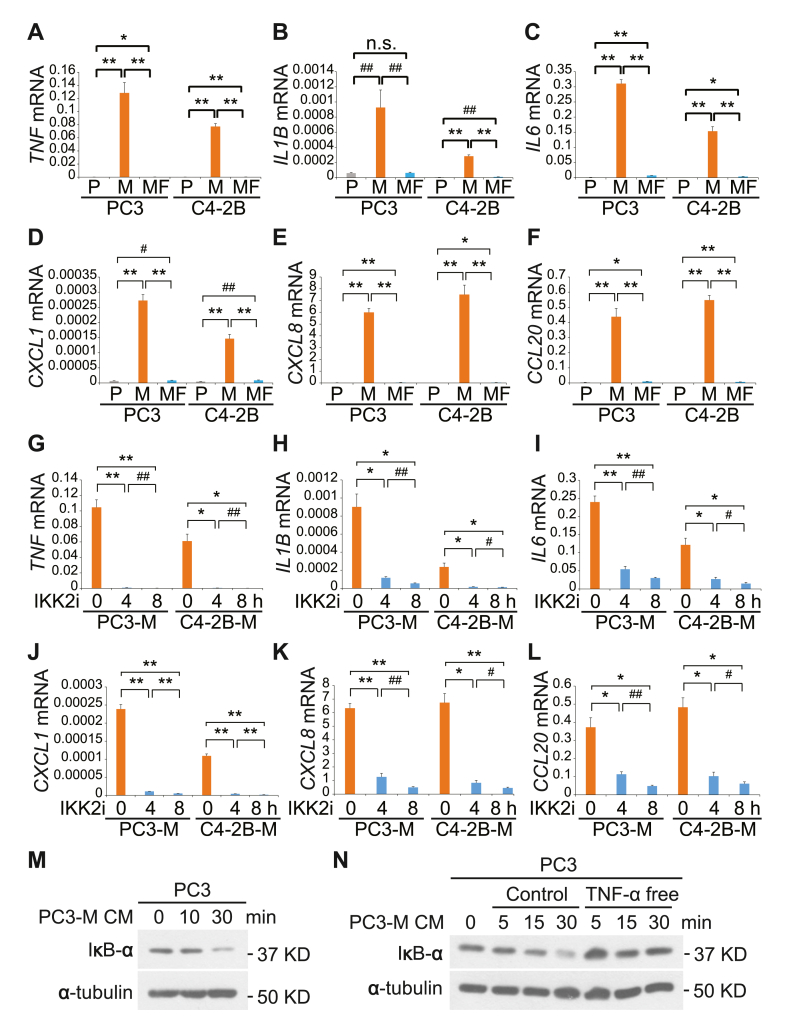
M. hyorhinis-induced TNF-α elicits cytokine/chemokine gene expression through NF-κB activation. (A–F), mRNA levels of the indicated genes in the parental (PC3–P and C4–2B–P), M. hyorhinis-infected (PC3-M and C4–2B-M), and previously infected after elimination of M. hyorhinis (PC3-MF and C4–2B-MF) PCa cells were quantified by qRT-PCR. (G–L), PC3-M and C4–2B-M cells were treated with 1 μM IKK2 inhibitor VI (IKK2i) for the indicated time and mRNA levels of the indicated genes were quantified by qRT-PCR. All results represent mean ± SD values from triplicate assays, and the experiments were repeated three times. #P < 0.05, ##P < 0.01, *P < 0.001, **P < 0.0001, n.s., not significant. (M), Western blot analysis of IκB-α expression in PC3 cells after applying PC3-M CM for the indicated time. (N), Western blot analysis of IκB-α expression in PC3 cells after applying PC3-M CM or TNF-α free PC3-M CM for the indicated time. α-Tubulin was used as a loading control. See full images in Supplementary Figure S5. PC3-M CM, PC3-M culture medium.

Next, the mRNA expression profile of the genes co-expressed in high *TNF* mRNA expressing patient groups (Data resource: TCGA, PanCancer Atlas; # groups (altered/all): 20/492) was generated using cBioPortal analysis (www.cbioportal.org). Because the genes with elevated expression in high *TNF* mRNA expressing patient groups were cytokines and chemokines (Table S2), gene profiling was conducted to further investigate the detailed mechanism regulated by *M. hyorhinis*-induced TNF-α in PCa cells. In parallel with TNF-α secretion levels in PC3-M and C4–2B-M cells (Fig. 1C and D), we found that mRNA levels of interleukin 1 beta (*IL1B*), interleukin 6 (*IL6*), C-X-C motif chemokine ligand 1 (*CXCL1*), *CXCL8*/*IL8*, and C–C motif chemokine ligand 20 (*CCL20*), as well as TNF-α (*TNF*) were all significantly induced in *M. hyorhinis* infected PCa cells compared to the parental cell lines (Fig. 4A–F). However, mRNA levels of those genes were attenuated in PC3-MF and C4–2B-MF cells (Fig. 4A–F). These cytokines and chemokines had been previously reported to be associated with tumorigenesis and progression of several tumors, including prostate cancer [[32], [33], [34], [35], [36], [37], [38], [39]]. Unlike co-expression data in the high TNF mRNA group in PCa patients (Table S2), the gene expression of other cytokines/chemokines or their receptors was not changed or significantly elevated in PC3-M and C4–2B-M cells compared to the parental cell lines (qRT-PCR data not shown; cytokines/chemokines: IL17A, CXCL3, CXCL6, CXCL17, CXCL10, CXCL9, CXCL12, CXCL13, CCL2, CCL8, CCL4L1, CCL5, CCL19, CCL21, CCL7, and CCL18; cytokine/chemokine receptors: TNFRSF1A/TNFR1, TNFRSF1B/TNFR2, CXCR6, CXCR3, CXCR5, CXCR4, CXCR2, CXCR1, CCR5, CCR1, CCR6, CCR7, CCR2, CCR9, and CCR3). Interestingly, we observed that the inhibition of NF-κB using the IKK2 inhibitor VI (IKK2i) in PC3-M and C4–2B-M cells significantly reduced *TNF* mRNA levels, indicating that NF-κB pathways may play an important role in modulating *M. hyorhinis*-induced TNF-α expression in PCa cells (Fig. 4G). As expected, the inhibition of NF-κB using IKK2i in PC3-M and C4–2B-M cells significantly reduced mRNA levels of *IL1B*, *IL6*, *CXCL1*, *CXCL8*/*IL8*, and *CCL20* in a time-dependent manner (Fig. 4H-L).

Since activation of NF-κB is a key event in TNF receptor 1 (TNFR1) signaling [16,21,23,40], further experiments were conducted to determine the extent to which *M. hyorhinis*-induced TNF-α from PCa would lead to NF-κB activation. First, the supernatant of culture medium of PC3-M cells (PC3-M CM) was collected and introduced to PC3 cells for 0, 10, and 30 min, respectively. Western blot analysis revealed that IκB-α levels were decreased in PC3 cells treated with PC3-M CM in a time dependent manner (Fig. 4M). Because IκB-α is a well-established negative regulator of the NF-κB activation pathway [41], our findings indicate that PC3-M cells secrete a molecule, or molecules, that initiates NF-kB activation in PC3 cells. Next, to investigate whether *M. hyorhinis*-induced TNF-α from PC3-M cells plays a specific role in NF-κB activation, we performed immunodepletion of TNF-α using anti-TNF-α antibody with protein A/G agarose beads in PC3-M CM. When PC3 cells were treated with PC3-M CM or PC3-M CM in which TNF-α was removed, there was no significant reduction in IκB-α level in PC3 cells treated with PC3-M CM without TNF-α (Fig. 4N). These data suggest *M. hyorhinis*-induced TNF-α from PC3-M cells is required for NF-κB activation in PC3 cells rather than other cytokines and chemokines.

### 3.5 NF-κB inhibition induces apoptosis in *M. hyorhinis*-infected PCa cells

**Fig. 5:**
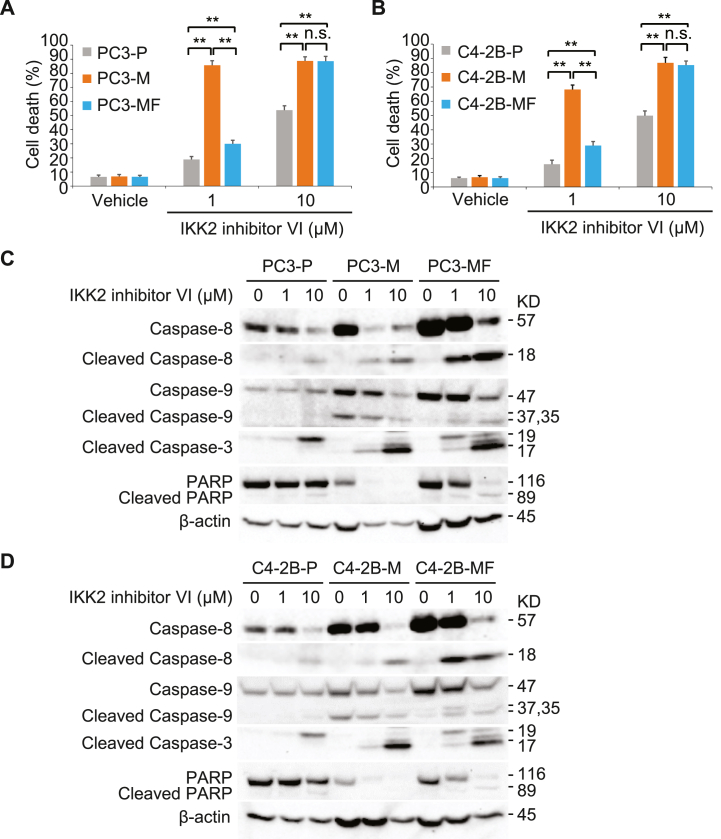
NF-κB inhibition induces apoptosis in M. hyorhinis infected PCa cells. (A), Cell death analysis in PC3–P, PC3-M, and PC3-MF cells treated with 1 μM and 10 μM IKK2 inhibitor VI for 48 h. (B), Cell death analysis in C4–2B–P, C4–2B-M, and C4–2B-MF cells treated with 1 μM and 10 μM IKK2 inhibitor VI for 48 h. Cell death was measured by trypan blue assay. All results represent mean ± SD values from triplicate assays, and the experiments were repeated three times. **P < 0.0001, n.s., not significant. (C), Western blot analysis using Caspase-8, cleaved Caspase-8, Caspase-9, cleaved Caspase-3, and PARP antibodies in PC3–P, PC3-M, and PC3-MF cells treated with 1 μM and 10 μM IKK2 inhibitor VI for 48 h. (D), Western blot analysis using Caspase-8, cleaved Caspase-8, Caspase-9, cleaved Caspase-3, and PARP antibodies in C4–2B–P, C4–2B-M, and C4–2B-MF cells treated with 1 μM and 10 μM IKK2 inhibitor VI for 48 h. β-Actin was used as a loading control. See full images in Supplementary Figure S6. (For interpretation of the references to colour in this figure legend, the reader is referred to the Web version of this article.)

Following the observation that *M. hyorhinis*-induced TNF-α elicited pro-survival NF-κB activation in PC3 cells, we sought to determine whether inhibition of the NF-κB pathway leads to PCa cell death. PC3, PC3-M, and PC3-MF cells were treated with 1 μM and 10 μM of IKK2i for 48 h, respectively. We observed that both low and high concentrations of IKK2i induced cell death in more than 80 % of PC3-M cells (Fig. 5A). However, PC3-MF cells induced relatively low cell death when treated with the low concentration of IKK2i, revealing that, at low concentration, IKK2 inhibition only affected apoptosis of the PC3 cells in which TNF-α was highly expressed. The same phenomenon was observed in C4–2B, C4–2B-M, and C4–2B-MF cells (Fig. 5B). Western blot analysis revealed that low concentrations of IKK2i did not result in caspase activation in the parental PC3 cells while high concentrations of IKK2i induced the partial cleavage of caspase-8 (18 kD), caspase-9 (35 kD), caspase-3 (19 kD), and PARP (89 kD). Full length caspase-8 levels were significantly increased in both PC3-M and PC3-MF cells compared to the parental PC3 cells and IKK2i treatment resulted in caspase-8 cleavage (18 kD) at both low and high concentrations (Fig. 5C). We also found an accumulation of high levels of cleaved caspase-9 without subsequent cleaved caspase-3 in non-treated PC3-M cells, but not in non-treated PC3-MF cells. This was observed despite the findings that full length caspase-9 levels were increased in both PC3-M and PC3-MF cells compared to the parental PC3 cells (Fig. 5C). However, in PC3-M cells, IKK2i treatment resulted in caspase-3 cleavage (17 kD) and PARP cleavage at both low and high concentrations (Fig. 5C). Like the findings in cell death assays, low concentrations of IKK2i showed decreased sensitivity of caspase-3 cleavage (17 kD) and PARP cleavage in PC3-MF cells compared to PC3-M cells. A similar trend of caspase activation was observed in C4–2B, C4–2B-M, and C4–2B-MF cells (Fig. 5D). Here, our findings demonstrated that aberrant caspase-9 activity in high TNF-α expressing *M. hyorhinis*-infected PCa cells did not result in caspase-3 cleavage and apoptosis. High concentrations of NF-κB inhibitor effectively induced cell death in *M. hyorhinis*-infected PCa cells by activating caspase-3 and PARP cleavage.

### 3.6 SMAC mimetics induce apoptosis of *M. hyorhinis*-infected PCa cells with altered TNF-α expression

**Fig. 6:**
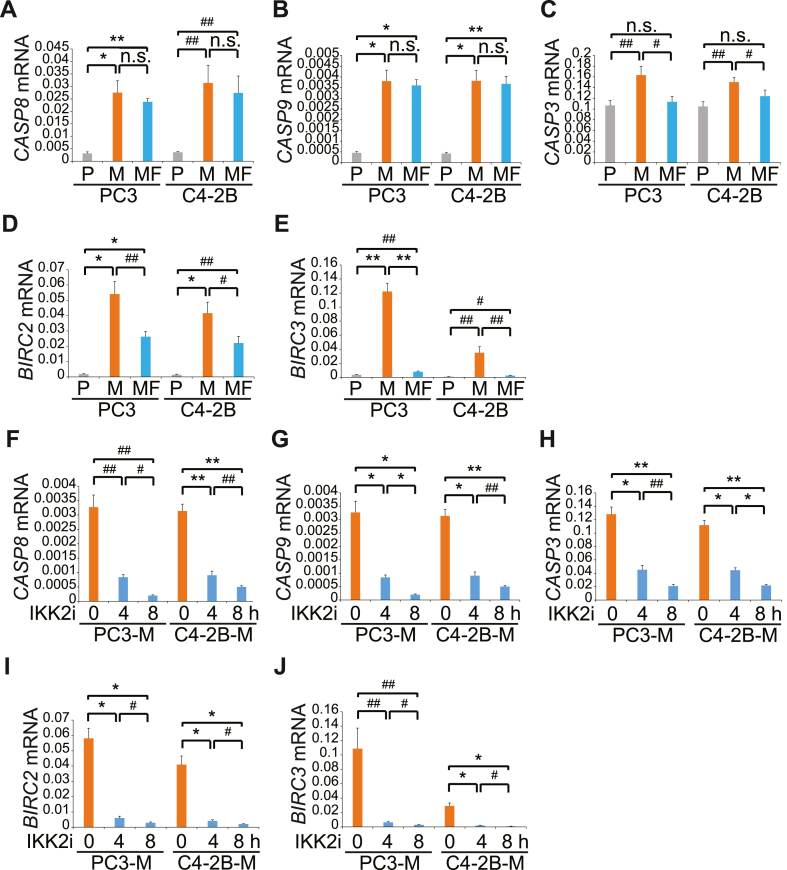
M. hyorhinis infection induces the gene expression of Caspase-8, Caspase-9, c-IAP1, and cIAP2 through NF-κB activation. (A–E), mRNA levels of the indicated genes in the parental (PC3–P and C4–2B–P), M. hyorhinis-infected (PC3-M and C4–2B-M), and previously infected after elimination of M. hyorhinis (PC3-MF and C4-2BMF) PCa cells were quantified by qRT-PCR. (F–J), PC3-M and C4–2B-M cells were treated with 1 μM IKK2 inhibitor VI for the indicated time and mRNA levels of the indicated genes were quantified by qRT-PCR. All results represent mean ± SD values from triplicate assays, and the experiments were repeated three times. #P < 0.05, ##P < 0.01, *P < 0.001, **P < 0.0001, n.s., not significant. Gene symbol: CASP8, Caspase-8; CASP9, Caspase-9; CASP3, Caspase-3; BIRC2, c-IAP1; BIRC3, c-IAP2.

It has previously been reported that cellular inhibitor of apoptosis protein 1 (c-IAP1, also named baculoviral IAP repeat-containing protein 2, BIRC2) and cellular inhibitor of apoptosis protein 2 (c-IAP2, also named baculoviral IAP repeat-containing protein 3, BIRC3) bind to pro-caspase-9 to inhibit the proteolytic processing of pro-caspase-3 [42]. To further investigate the possible relationship between the aberrant expression of caspase-9 and c-IAPs expression in PCa cells infected with *M. hyorhinis*, qRT-PCR was performed. We found that *M. hyorhinis* infection caused a significant elevation of *CASP8* (caspase-8) and *CASP9* (caspase-9) mRNA expression in PC3-M and C4–2B-M cells, and the levels were maintained in PC3-MF and C4–2B-MF cells regardless of the presence of *M. hyorhinis* (Fig. 6A and B). *CASP3* (Caspase-3) mRNA levels were slightly elevated during *M. hyorhinis* infection and decreased once *M. hyorhinis* was removed (Fig. 6C). Interestingly, significantly increased mRNA levels of *BIRC2* (c-IAP1) and *BIRC3* (c-IAP2) were observed when PC3 and C4–2B cells were infected with *M. hyorhinis* (Fig. 6D and E), suggesting that *M. hyorhinis* infection prevents PCa cells from undergoing high TNF-α-mediated cell death by modulating *BIRC2* and *BIRC3* expression. This is an important finding because *M. hyorhinis*-infected cells should be more prone to apoptosis due to high caspase-8 and caspase-9 expression. Treatment with NF-kB inhibitor, IKK2i, decreased mRNA levels of *CASP8*, *CASP9*, *CASP3*, *BIRC2*, and *BIRC3* in a time-dependent manner in PC3-M and C4–2B-M cells, showing that caspase-8/9 and its downstream cascade effector caspase-3, and BIRC2/3 expression are dependent on NF-κB activity (Fig. 6F–J).

**Fig. 7:**
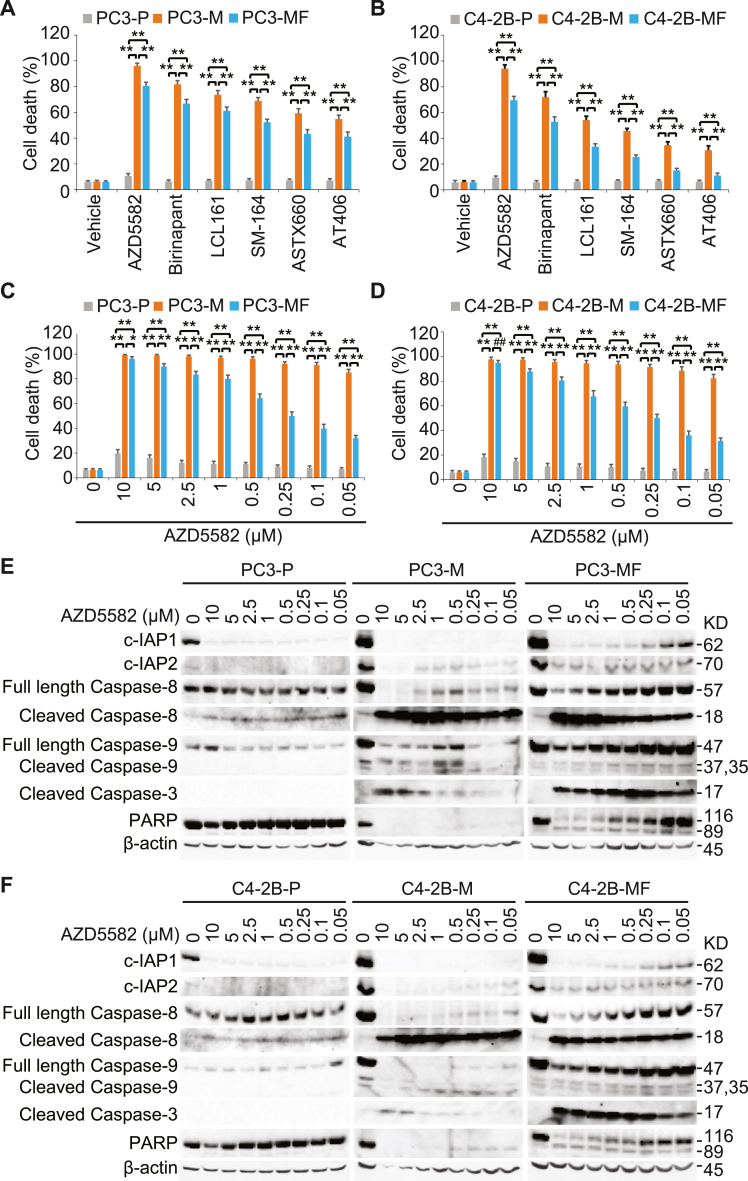
SMAC mimetic treatment selectively induced cell death of M. hyorhinis-infected PCa cells. (A), Cell death analysis in PC3–P, PC3-M, and PC3-MF cells treated with 1 μM of the indicated SMAC mimetics for 48 h. (B), Cell death analysis in C4–2B–P, C4–2B-M, and C4–2B-MF cells treated with 1 μM of the indicated SMAC mimetics for 48 h. (C), Cell death analysis in PC3–P, PC3-M, and PC3- MF cells treated with the indicated concentration of AZD5582 for 48 h. (D), Cell death analysis in C4–2B–P, C4–2B-M, and C4–2B-MF cells treated with the indicated concentration of AZD5582 for 48 h. Cell death was measured by trypan blue assay. All results represent mean ± SD values from triplicate assays, and the experiments were repeated three times. ##P < 0.01, *P < 0.001, **P < 0.0001. (E), Western blot analysis using c-IAP1, c-IAP2, Caspase-8, cleaved Caspase-8, Caspase-9, cleaved Caspase-3, and PARP antibodies in PC3–P, PC3-M, and PC3-MF cells treated with the indicated concentration of AZD5582 for 48 h. See full images in Supplementary Figure S7. (F), Western blot analysis using c-IAP1, c-IAP2, Caspase-8, cleaved Caspase-8, Caspase-9, cleaved Caspase-3, and PARP antibodies in C4–2B–P, C4–2B-M, and C4–2B-MF cells treated with the indicated concentration of AZD5582 for 48 h. β-Actin was used as a loading control. See full images in Supplementary Figure S8. (For interpretation of the references to colour in this figure legend, the reader is referred to the Web version of this article.)

SMAC mimetics are a contemporary class of drugs designed to treat solid tumors such as PCa. These small molecules work by targeting the inhibitor of apoptosis protein (IAP) family [43]. We found that *BIRC2* and *BIRC3* mRNA levels were highly up-regulated in PC3-M and C4–2B-M cells (Fig. 6D and E), suggesting SMAC mimetics may promote apoptosis in *M. hyorhinis*-infected PCa cells. To test the sensitivity of PCa to IAP inhibition, cell death analysis was performed using synthetic SMAC mimetics, including AZD5582, Birinapant, LCL161, SM-164, ASTX660, and AT406. SMAC mimetics (1 μM) were treated in PC3, PC3-M, and PC3-MF cells for 48 h. The data revealed that all synthetic SMAC mimetics induced significant cell death in PC3-M and PC3-MF cells, but led to higher cell death in PC3-M cells compared to PC3-MF cells (Fig. 7A). SMAC mimetics also elevated cell death in C4–2B-M and C4–2B-MF cells, but higher cell death in C4–2B-M cells compared to C4–2B-MF cells (Fig. 7B). Based on these findings, further analyses were conducted with AZD5582, which exhibited the most potency in PCa cells (Fig. 7A and B). AZD5582 is a synthetic compound that binds to the BIR3 domain of IAPs to cause c-IAP1 degradation and has been shown to induce an anti-proliferative effect in cancer cell lines [44]. AZD5582 treatment for 48 h produced more than 80 % cell death in both PC3-M and C4–2B-M cells regardless of the concentration, while AZD5582 treatment in PC3-MF and C4–2B-MF cells induced cell death in a dose-dependent manner (Fig. 7C and D). Western blot analysis demonstrated that c-IAP1 and c-IAP2 levels were noticeably increased in PC3-M cells. Increased c-IAP1 and c-IAP2 levels were maintained in PC3-MF cells regardless of the presence of *M. hyorhinis*, in contrast to mRNA levels (Fig. 6D and E). AZD5582 treatment blocked c-IAP1 expression and significantly reduced c-IAP2 expression in PC3-M cells regardless of the concentration. AZD5582 treatment in PC3-MF cells blocked c-IAP1 expression in a dose-dependent manner and significantly reduced c-IAP2 expression regardless of the concentration. (Fig. 7E). AZD5582 treatment promoted caspase-8, caspase-9, and caspase-3 activation and subsequent PARP cleavage in both PC3-M and PC3-MF cells (Fig. 7E). Since high cell death occurred after AZD5582 treatment in PC3-M cells, the cleaved caspase-9 and -3 levels may not reflect their relative amount in cells that have already undergone apoptosis. However, enhanced active caspase-3-dependent PARP cleavage was nevertheless observed in all PC3-M cells treated with varying concentrations of AZD5582 (Fig. 7E). In addition, the aberrant expression of cleaved caspase-9 without subsequent downstream caspase-3 activation was observed again in control PC3-M cells. In PC3-MF cells, significant PARP cleavage was detected for AZD5582 concentrations higher than 1 μM. However, at concentrations lower than 1 μM, the effectiveness of AZD5582 was reduced, resulting in decreased PARP cleavage. Similar results were obtained when C4–2B, C4–2B-M, and C4–2B-MF cells were treated with different concentrations of AZD5582 (Fig. 7F). Together, these results revealed that the c-IAP antagonist AZD5582 may function as a potential therapeutic agent of *M. hyorhinis*-infected PCa.

## 4 Discussion

Mycoplasma is a gram-negative microbe with the size of 0.2–0.3 μm without a cell wall and has been postulated to be associated with the development and progression of several types of cancer [10,11,15,45,46]. Recent discoveries suggest a potential correlation between mycoplasma infection and prostate cancer development and progression [47]. Specifically, *M. hyorhinis* expression was found in 69 % of human prostatic tissues, and *M. hyorhinis* immunoreactivity has been associated with prostatic cancer advancement [48]. Additionally, persistent mycoplasma infection, including *M. hyorhinis*, has been shown to participate in malignant transformation of benign human prostate cells, promoting anchorage-independent growth, migration, and invasion *in vitro* and *in vivo* [9]. However, the role of mycoplasma as a causative or facilitative agent during tumor development and progression has yet to be determined. Here, we showed that *M. hyorhinis*-infected PCa cells secrete aberrant levels of TNF-α. In addition, TMA data indicated that a high incidence of *M. hyorhinis* infection is correlated with increased TNF-α in tissues of PCa patients. Interestingly, analysis from TCGA data (PanCancer Atlas) using cBioPortal also demonstrated a possible association between high levels of TNF-α and frequency of mycoplasma infection not only in prostate adenocarcinoma, but also in different types of tumors from the reproductive and urinary organs of males and females, such as uterine corpus endometrial carcinoma, bladder urothelial carcinoma, and ovarian serous cystadenocarcinoma (Table S1). Our analysis also showed that a higher degree of TNF-α was associated with greater lymph node metastasis and a higher degree of advanced stages of cancer in prostate adenocarcinoma, uterine corpus endometrial carcinoma, and bladder urothelial carcinoma (Fig. S2 and Fig. S3). These findings suggest that *M. hyorhinis*-induced TNF-α may play an important role in promoting tumor survival and progression in PCa.

TNF-α is a proinflammatory cytokine that has been implicated in tumor development, survival, malignancy [19,22,23]. TNF-α can function as both an autocrine and paracrine growth factor in ovarian cancer [23]. Likewise, chronic TNF-α exposure increases the metastatic potential of PCa via phosphorylation of extracellular signal-regulated kinase (ERK) leading to C–C chemokine receptor 7 (CCR7) upregulation [25]. Because TNF-α plays an important role in carcinogenesis and tumor progression, further studies on how *M. hyorhinis*-infected PCa cells induce expression and secretion of TNF-α may help elucidate the possible molecular mechanisms by which *M. hyorhinis* plays a role in PCa carcinogenesis. Numerous studies have reported that TNF-α activates NF-κB [[21], [22], [23],^40^]. Our data also demonstrated that aberrant secretion of TNF-α from PC3-M cells leads to NF-κB activation in parental PC3 cells. When TNF-α was withdrawn from the PC3-M cell conditioned medium, NF-κB activation was not observed in PC3 cells treated with this medium. These findings suggest that *M. hyorhinis*-induced TNF-α may play a key role in NF-κB activation to promote PCa cell survival and progression. TNF-α mediates its effect through two different receptors: TNF-α receptor I (TNFR1) and TNF-α receptor 2 (TNFR2) [22]. TNFR2 is specifically expressed on endothelial and immune cells, but TNFR1 is ubiquitously expressed on all cell types and has been reported to activate the NF-κB pathway leading to the expression of a variety of inflammation-related genes [22]. Moreover, TNF-α has been known to play a critical role in mediating chronic inflammation and facilitating tumor initiation and promotion by regulating the proliferation and survival of neoplastic cells while also affecting endothelial cells and other immune cells present in the tumor microenvironment [18]. Thus, understanding the role of *M. hyorhinis*-induced TNF-α in establishing the link between inflammation and cancer has the potential to support the development of an effective strategy for PCa therapy.

Results presented here demonstrate that high TNF-α expressing PC3-M and C4–2B-M cells produced increased levels of cytokines and chemokines, which have been previously reported to be regulated by the NF-κB pathway [49]. Treatment of NF-κB inhibitor in PC3-M and C4–2B-M cells blocked the expression of these cytokines and chemokines, suggesting TNF-α-mediated NF-κB activation is essential for PCa cells to alter the tumor environment and promote the advancement of PCa. Further, NF-κB inhibitor led to cell death in *M. hyorhinis*-infected PCa cells as well as PCa cells that were once infected by the *M. hyorhinis* at high concentrations through activation of the caspase cascade. These results suggest that targeting TNF-α-induced NF-κB signaling in mycoplasma-infected PCa cells may serve as a valid therapeutic strategy for PCa. However, to date, no conventional IKKβ/NF-κB-targeting inhibitors have progressed to clinical treatment due to the toxicities associated with the systemic inhibition of NF-κB [50,51]. Thus, an urgent need persists for the development of a more effective and selectively potent drug to therapeutically target pathological TNF-α and NF-κB signaling in *M. hyorhinis*-infected PCa.

Our data also demonstrate that *M. hyorhinis*-infected PCa cells express an aberrantly high level of cleaved caspase-9 accompanied by no caspase-3 activation. The inhibitors of apoptosis (IAP) are a family of proteins, and the human genome encodes eight members: NAIP (BIRC1), BIRC2 (c-IAP1), BIRC3 (c-IAP2), XIAP (BIRC4), BIRC5 (survivin), BIRC6 (apollon), BIRC7 (livin), and BIRC8 [52]. IAP family members contain at least one baculoviral IAP repeat (BIR) and are involved in cell division, proliferation, and the apoptosis pathways [52]. Among IAPs, c-IAP1 and c-IAP2 are known as direct inhibitors of caspases-3 and -7 [53]. In addition, IAPs are highly expressed in several malignancies, and they have been reported to block cell death via modulating both NF-κB signaling and TNF-α-dependent apoptosis [54,55]. Recently, the use of SMAC mimetics, small molecules that mimic an endogenous IAP antagonist known as SMAC, showed promising anti-tumor activity specific to cancer cells by promoting caspase activation [56,57]. Therefore, we screened multiple synthetic SMAC mimetics to evaluate their effects on PCa cells with elevated TNF-α as a result of *M. hyorhinis* infection. As expected, SMAC mimetics promoted cell death in aggressive *M. hyorhinis*-infected PCa cells and PCa cells that had previously been infected with *M. hyorhinis*. Furthermore, AZD5582 treatment resulted in the most potent cell death in *M. hyorhinis*-infected PCa cells amongst several SMAC mimetics examined and that AZD5582 treatment inhibited c-IAP1 and c-IAP2 and allowed caspase-3 activation, leading to cell death.

SMAC mimetics were developed as a cancer therapeutic reagent that antagonize IAPs and therefore inhibits proliferation and cancer cell survival [58]. According to the NIH U.S. National Library of Medicine for Clinical Trials, many SMAC mimetics, including Birinapant (Medivir), LCL161 (Novartis), AT406/Debio11143/Xevinapant (Merck), and ASTX660/Tolinapant (Astex) are currently under the phase I or II clinical trials for hematological and solid cancers [58]. The robust preclinical and clinical research revealed that TNF-α plays a crucial role in increasing the effectiveness of SMAC mimetics for cancer treatment, and various approaches have been developed to enhance the levels of TNF-α levels extrinsically through techniques such as Isolated Limb Perfusion or intrinsically by targeting parallel signaling pathways to enhance the sensitivity of SMAC mimetics to tumors [58]. In addition, the responders of a neoadjuvant trial using LCL161 in triple-negative breast cancers exhibited high TNF-α and RIPK1 levels [59]. Consistent with our findings, clinical trial results [59] also suggest that TNF-α levels may specify predictive sensitivity and potency to SMAC mimetics, indicating potential trial investigation for prostate cancer as well.

AZD5582 was originally developed as a cancer therapeutic reagent which would antagonize IAPs and therefore inhibit proliferation and cancer cell survival [44]. Recently, the use of AZD5582 has been broadened for potential human immunodeficiency virus (HIV) treatment strategies as well. AZD5582 treatment triggered latency reversal of systemic HIV and SIV and showed the potential for use of a combination treatment approach to aid in systemic clearance of persistent HIV infection in resting CD4^+^ T cells [60]. More importantly, it has been shown that 97 doses of AZD5582 treatment for twelve SIV-infected *Rhesus macaques* were well-tolerated, and only two instances resulted in mild adverse reactions, demonstrating that AZD5582 has minimal side effects with little toxicity *in vivo* [60]. Despite the pivotal role played by NF-κB pathways, inhibition of this pathway is often associated with adverse drug reactions due to the lack of specificity of small molecules [43]. Thus, the use of AZD5582 and other SMAC inhibitors examined in this study to specifically target IAPs induced by high NF-κB activity may function as a reliable and effective therapeutic strategy to promote cancer cell death in malignant PCa patients.

In our study, chronic *M. hyorhinis* infection promotes the advancement of PCa via aberrant TNF-α secretion, and findings from this study provide a further understanding of mycoplasma-oncogenesis. Our compatible results from cancer cell lines and tissue microarrays of patients infected with *Mycoplasma* are consistent with others' findings and propose the importance of postulating novel approaches for prevention, diagnosis, and therapeutic regimen, such as SMAC mimetics, to treat prostate cancers. However, because immortalized cell lines exhibit limitations as preclinical models, further validation and elucidation of mechanisms with *in vivo* models using xenograft mice may be relevant in the design of further studies. In conclusion, our data suggest that TNF-α acts as a major mediator of *M. hyorhinis*-induced malignant PCa cell progression. Targeting *M. hyorhinis*-infected PCa with SMAC mimetics may represent a novel therapeutic strategy for PCa patients.

## CRediT authorship contribution statement

**Jin Koo Kim:** Conceptualization, Data curation, Formal analysis, Investigation, Methodology, Resources, Validation, Visualization, Writing – original draft, Writing – review & editing. **Insoon Chang:** Formal analysis, Writing – original draft, Writing – review & editing. **Younghun Jung:** Formal analysis, Investigation, Writing – review & editing. **Zach Kaplan:** Formal analysis, Software, Writing – review & editing. **Elliott E. Hill:** Formal analysis, Resources, Supervision, Writing – review & editing. **Russell S. Taichman:** Formal analysis, Funding acquisition, Resources, Supervision, Writing – review & editing. **Paul H. Krebsbach:** Conceptualization, Formal analysis, Project administration, Resources, Supervision, Validation, Writing – original draft, Writing – review & editing.

## Declaration of competing interest

The authors declare that they have no known competing financial interests or personal relationships that could have appeared to influence the work reported in this paper.
