# Supplementary material for: Mycoplasma hyorhinis infection promotes TNF-α signaling and SMAC mimetic-mediated apoptosis in human prostate cancer
Source: Heliyon. 2023 Oct 11;9(10):e20655. doi: 10.1016/j.heliyon.2023.e20655 (PMC10585237; doi:10.1016/j.heliyon.2023.e20655)
Supplement: Multimedia component 1 [file mmc1.docx]

**Supporting Information**

***Mycoplasma hyorhinis* infection promotes TNF-α signaling and SMAC mimetic-mediated apoptosis in human prostate cancer**

Jin Koo Kim^a^, Insoon Chang^b^, Younghun Jung^c,e^, Zach Kaplan^c^, Elliott E. Hill^d^, Russell S. Taichman^c,f^, and Paul H. Krebsbach^a,^*

^a^ Division of Oral and Systemic Health Sciences, University of California, Los Angeles School of Dentistry, Los Angeles, California, USA

^b^ Section of Endodontics, University of California, Los Angeles School of Dentistry, Los Angeles, California, USA

^c^ Department of Periodontics and Oral Medicine, University of Michigan School of Dentistry, Ann Arbor, Michigan, USA

^d^ Department of Biologic and Materials Sciences, University of Michigan School of Dentistry, Ann Arbor, Michigan, USA

^e^ Department of Neurology, Boston University School of Medicine, Boston, Massachusetts, USA

^f^ Department of Periodontics, University of Alabama at Birmingham, Birmingham, Alabama, USA.

*To whom correspondence should be addressed: Paul H. Krebsbach: University of California, Los Angeles School of Dentistry, Los Angeles, California 90095; E-mail: [pkrebsbach@dentistry.ucla.edu](mailto:pkrebsbach@dentistry.ucla.edu)

**Contents**

**Table S1.** Mycoplasma microbiome signature in high *TNF* mRNA group in patients with prostate

adenocarcinoma or the different types of tumors.

**Table S2.** The genes of cytokines and chemokines co-expressed in high *TNF* mRNA group in patients

with prostate adenocarcinoma.

**Table S3.** Primers used for qRT-PCR.

**Figure S1.** *M. hyorhinis* induces the expression of *TNF* mRNA and TNF-α protein.

**Figure S2.** Clinical data analysis between high *TNF* mRNA (Altered) group and unaltered group in

patients with prostate adenocarcinoma.

**Figure S3.** Clinical data analysis between high *TNF* mRNA (Altered) group and unaltered group in

patients with the different types of tumors.

**Figure S4.** Full images of Figure 1A, Figure 1C, and Figure 1D.

**Figure S5.** Full images of Figure 4M and Figure 4N.

**Figure S6.** Full images of Figure 5C and Figure 5D.

**Figure S7.** Full images of Figure 7E.

**Figure S8.** Full images of Figure 7F.

**Figure S9.** Full images of Figure S1B.

**Table S1.** Mycoplasma microbiome signature in high *TNF* mRNA group in patients with prostate

adenocarcinoma or the different types of tumors.

| Cancer name | Log ratio | p-Value | % Altered group | # groups (Altered\All) |
| --- | --- | --- | --- | --- |
| **Prostate Adenocarcinoma** | 0.17 | 0.0334** | 4 | 20\492 |
| **Uterine Corpus Endometrial Carcinoma** | 0.4 | 0.0143** | 4.4 | 23\523 |
| **Pancreatic Adenocarcinoma** | 0.24 | 0.0338** | 4 | 7\177 |
| **Bladder Urothelial Carcinoma** | 0.33 | 0.0631* | 2 | 8\407 |
| **Ovarian Serous Cystadenocarcinoma** | 0.26 | 0.0537* | 5.7 | 17\300 |
| Mesothelioma | 0.25 | 0.0916 | 3.4 | 3\87 |
| Adrenocortical Carcinoma | 0.1 | 0.43 | 6.4 | 5\78 |
| Liver Hepatocellular Carcinoma | 0.09 | 0.295 | 3.9 | 14\358 |
| Testicular Germ Cell Tumors | 0.09 | 0.542 | 6 | 8\133 |
| Kidney Renal Clear Cell Carcinoma | 0.05 | 0.495 | 4.5 | 23\509 |
| Lung Squamous Cell Carcinoma | 0.05 | 0.61 | 1.1 | 5\471 |
| Head and Neck Squamous Cell Carcinoma | 0.02 | 0.721 | 2.5 | 13\514 |
| Cervical Squamous Cell Carcinoma | 0.01 | 0.967 | 2 | 6\294 |
| Sarcoma | -0.01 | 0.9 | 4.7 | 12\253 |
| Skin Cutaneous Melanoma | -0.01 | 0.952 | 1.36 | 6\441 |
| Brain Lower Grade Glioma | -0.02 | 0.706 | 2.1 | 11\513 |
| Breast Invasive Carcinoma | -0.02 | 0.719 | 5.3 | 57\1070 |
| Lung Adenocarcinoma | -0.04 | 0.608 | 1 | 5\491 |
| Thyroid Carcinoma | -0.05 | 0.507 | 2.6 | 13\497 |
| Stomach Adenocarcinoma | -0.08 | 0.635 | 3.6 | 15\409 |
| Thymoma | -0.08 | 0.688 | 3.4 | 4\119 |
|  |  |  |  |  |

Data resource: TCGA, PanCancer Atlas. Student’s *t*-test; **p* < 0.064, ***p* < 0.05.

**Table S2.** The genes of cytokines and chemokines co-expressed in high *TNF* mRNA group in patients

with prostate adenocarcinoma.

| Co-expressed gene symbol | Spearman's correlation | p-Value |
| --- | --- | --- |
| (cytokine/chemokine) |  | (by 2-sided t-test) |
| **IL1B*** | 0.719 | 1.68E-79 |
| **IL6*** | 0.53 | 4.86E-37 |
| **IL8/CXCL8*** | 0.526 | 2.12E-36 |
| IL17A | 0.347 | 2.34E-15 |
| CXCL3 | 0.598 | 4.34E-49 |
| **CXCL1*** | 0.577 | 5.35E-45 |
| CXCL6 | 0.419 | 2.29E-22 |
| CXCL17 | 0.345 | 3.14E-15 |
| CXCL10 | 0.301 | 8.74E-12 |
| CXCL9 | 0.279 | 2.92E-10 |
| CXCL12 | 0.278 | 3.32E-10 |
| CXCL13 | 0.23 | 2.56E-07 |
| CCL2 | 0.589 | 3.13E-47 |
| CCL8 | 0.575 | 1.35E-44 |
| CCL4L1 | 0.56 | 6.56E-42 |
| CCL5 | 0.442 | 5.76E-25 |
| **CCL20*** | 0.428 | 2.26E-23 |
| CCL19 | 0.416 | 4.78E-22 |
| CCL21 | 0.304 | 5.65E-12 |
| CCL7 | 0.287 | 8.32E-11 |
| CCL18 | 0.255 | 1.03E-08 |
| (cytokine/chemokine receptor) | Spearman's correlation | p-Value |
| TNFRSF1A/TNFR1 | 0.247 | 2.84E-08 |
| TNFRSF1B/TNFR2 | 0.549 | 4.67E-40 |
| CXCR6 | 0.505 | 3.58E-33 |
| CXCR3 | 0.482 | 5.00E-30 |
| CXCR5 | 0.472 | 1.10E-28 |
| CXCR4 | 0.456 | 1.19E-26 |
| CXCR2 | 0.394 | 1.09E-19 |
| CXCR1 | 0.286 | 1.10E-10 |
| CCR5 | 0.594 | 3.63E-48 |
| CCR1 | 0.57 | 1.13E-43 |
| CCR6 | 0.53 | 5.40E-37 |
| CCR7 | 0.53 | 5.98E-37 |
| CCR2 | 0.486 | 1.44E-30 |
| CCR9 | 0.305 | 5.11E-12 |
| CCR3 | 0.263 | 3.37E-09 |

Data resource: TCGA, PanCancer Atlas. # groups (Altered\All): 20\492. * Data were shown in Figure 4.

**Table S3.** Primers used for qRT-PCR.

| Primer name | Sequence (5'-3') | Primer used for figure |
| --- | --- | --- |
| TNF-Forward (F) | AGGCAGTCAGATCATCTTC | Yes |
| TNF-Reverse (R) | TTATCTCTCAGCTCCACG | Yes |
| IL1B-F | CTAAACAGATGAAGTGCTCC | Yes |
| IL1B-R | GGTCATTCTCCTGGAAGG | Yes |
| IL6-F | GCAGAAAAAGGCAAAGAATC | Yes |
| IL6-R | CTACATTTGCCGAAGAGC | Yes |
| CXCL1-F | ATGCTGAACAGTGACAAATC | Yes |
| CXCL1-R | TCTTCTGTTCCTATAAGGGC | Yes |
| CXCL8/IL8-F | GTTTTTGAAGAGGGCTGAG | Yes |
| CXCL8/IL8-R | TTTGCTTGAAGTTTCACTGG | Yes |
| CCL20-F | TATATTGTGCGTCTCCTCAG | Yes |
| CCL20-R | GCTATGTCCAATTCCATTCC | Yes |
| CASP8-F | CTACAGGGTCATGCTCTATC | Yes |
| CASP8-R | ATTTGGAGATTTCCTCTTGC | Yes |
| CASP9-F | CTCTACTTTCCCAGGTTTTG | Yes |
| CASP9-R | TTTCACCGAAACAGCATTAG | Yes |
| CASP3-F | AAAGCACTGGAATGACATC | Yes |
| CASP3-R | CGCATCAATTCCACAATTTC | Yes |
| BIRC2-F | CCAACAGAAGATGTTTCAGG | Yes |
| BIRC2-R | ATTATACCCCTGCAAATAGGG | Yes |
| BIRC3-F | ACAAGCAAGAGAACTGATTG | Yes |
| BIRC3-R | GATCTGAAACATCTTCTGTGG | Yes |
| GAPDH-F | TCGGAGTCAACGGATTTG | Yes |
| GAPDH-R | CAACAATATCCACTTTACCAGAG | Yes |
| Primer name | Sequence (5'-3') | Primer used for figure |
| TNFRSF1A-F | CCCCTGGTCATTTTCTTTG | NO |
| TNFRSF1A-R | ATTTCCCACAAACAATGGAG | NO |
| TNFRSF1B-F | ACCAGGTGGAAACTCAAG | NO |
| TNFRSF1B-R | TTTCAGTTCCTGGTCTGG | NO |
| CXCR1-F | TTAAGTCACTCTGATCTCTGAC | NO |
| CXCR1-R | TGGTTTGATCTAACTGAAGC | NO |
| CXCR2-F | CCAGTCAGGATTTAAGTTTACC | NO |
| CXCR2-R | GTTGATTTCCAGGGATTCTG | NO |
| CXCR3-F | GTCCTTGAGGTGAGTGAC | NO |
| CXCR3-R | TCTCCATAGTCATAGGAAGAG | NO |
| CXCR4-F | AACTTCAGTTTGTTGGCTG | NO |
| CXCR4-R | GTGTATATACTGATCCCCTCC | NO |
| CXCR5-F | AGTATCCTCATTTGGGGTAG | NO |
| CXCR5-R | GCATTGGATGATTAGGATGG | NO |
| CXCR6-F | GGTGTTCATCAGAACAGAC | NO |
| CXCR6-R | GAAAGACCTTGCTGAACTG | NO |
| CCR1-F | CCTTGGAACCAGAGAGAAG | NO |
| CCR1-R | AATACCAAGGAGTACAGAGG | NO |
| CCR2-F | AAGCCTTTTTCACATAGCTC | NO |
| CCR2-R | CTTTCACATTCTTTCCTGGTC | NO |
| CCR3-F | CACTGCTGAGTTGTATTGG | NO |
| CCR3-R | GCTCTGGTATCAGCTTTTTC | NO |
| CCR5-F | TATTCTGTGTAGTGGGATGAG | NO |
| CCR5-R | TGCTGTTTCTTTTGAAGGAG | NO |
| CCR6-F | TATGCTGGTGAACAGAAATG | NO |
| CCR6-R | CTCAATAAAGAAGGAGCTGTC | NO |
| CCR7-F | TTGTCATTTTCCAGGTATGC | NO |
| CCR7-R | AATGATGGAGTACATGATAGGG | NO |
| CCR9-F | GACTAACACAAGCCCTATTC | NO |
| CCR9-R | CACAGTAGAAGTCAGTGAAG | NO |
| Primer name | Sequence (5'-3') | Primer used for figure |
| IL17A-F | CATTGGTGTCACTGCTAC | NO |
| IL17A-R | TCGGTTGTAGTAATCTGAGG | NO |
| CXCL3-F | CCTCAAGAACATCCAAAGTG | NO |
| CXCL3-R | CCCCTTGTTCAGTATCTTTTC | NO |
| CXCL6-F | CCTCTCTTGACCACTATGAG | NO |
| CXCL6-R | GTTTTGGGGTTTACTCTCAG | NO |
| CXCL9-F | AGGTCAGCCAAAAGAAAAAG | NO |
| CXCL9-R | TGAAGTGGTCTCTTATGTAGTC | NO |
| CXCL10-F | AAAGCAGTTAGCAAGGAAAG | NO |
| CXCL10-R | TCATTGGTCACCTTTTAGTG | NO |
| CXCL12-F | AGAAAGCTTTAAACAAGGGG | NO |
| CXCL12-R | AGCCTTTCTCTTCTTCTGTC | NO |
| CXCL13-F | CATAGTCTGGAAGAAGAACAAG | NO |
| CXCL13-R | AAGAATGCAGGTGTTCTTAG | NO |
| CXCL16-F | CGGTGTCTATACTACACGAG | NO |
| CXCL16-R | TCAAGACAGCTCATCAATTC | NO |
| CXCL17-F | AGAAAACAAGACACCAAAGG | NO |
| CXCL17-R | GTTTAATTGGAAGAGTGGGC | NO |
| CCL2-F | AGACTAACCCAGAAACATCC | NO |
| CCL2-R | ATTGATTGCATCTGGCTG | NO |
| CCL4L1-F | CCTCTGAGAAAACCTCTTTG | NO |
| CCL4L1-R | TGGTCTCATAGTAATCTACCAC | NO |
| CCL5-F | AAGTCTCTAGGTTCTGAGC | NO |
| CCL5-R | TTTTATGGTTGCATTGAGAAC | NO |
| CCL7-F | AATCTTCAAGACCAAACTGG | NO |
| CCL7-R | TTGGAGTTTGGGTTTTCTTG | NO |
| CCL8-F | CTCCAAGATGAAGGTTTCTG | NO |
| CCL8-R | ATTGATCACGTTAAAGCAGC | NO |
| CCL18-F | CTATACCTCCTGGCAGATTC | NO |
| CCL18-R | CTCTCTTGGTTAGGAGGATG | NO |
| CCL19-F | ACCTCAGCCAAGATGAAG | NO |
| CCL19-R | TAATTCACAATGCTTGACTCG | NO |
| CCL21-F | AGCTATCCTGTTCTTGCC | NO |
| CCL21-R | TTCTTGCCAGTCTTGGAG | NO |
|  |  |  |
|  |  |  |


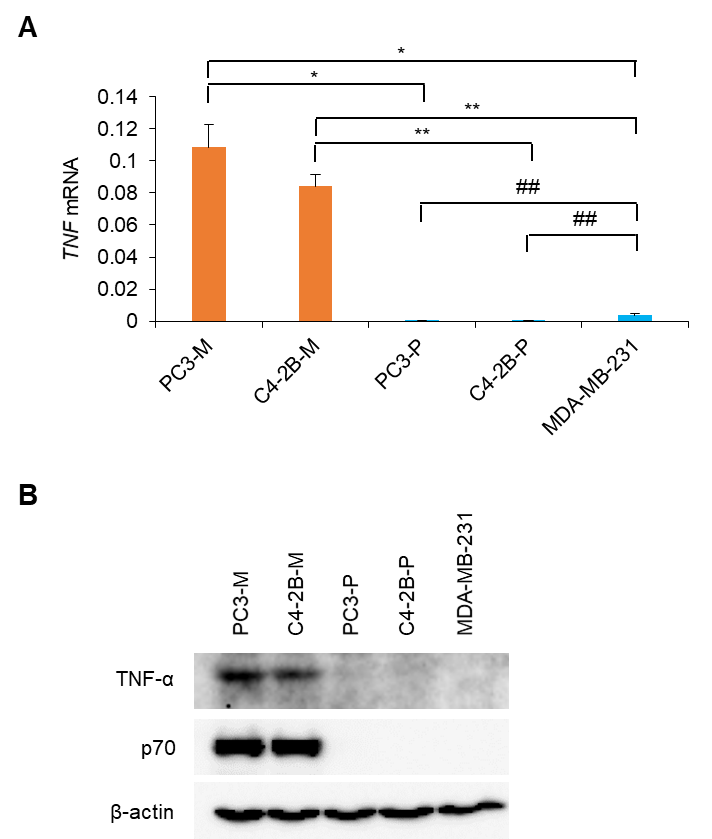


25

20

45

70

**Figure S1.** *M. hyorhinis* induces the expression of *TNF* mRNA and TNF-α protein. **(A)**, *TNF* mRNA levels in *M. hyorhinis*-infected (PC3-M and C4-2B-M), parental (PC3-P and C4-2B-P), and MDA-MB-231 cells were quantified by qRT-PCR. All results represent mean ± SD values from triplicate assays, and the experiments were repeated three times. **##***P* < 0.01, **P* < 0.001, ***P* < 0.0001. **(B)**, Western blot analysis of TNF-α expression in *M. hyorhinis*-infected (PC3-M and C4-2B-M), parental (PC3-P and C4-2B-P), and MDA-MB-231 cells. β-actin was used as a loading control. See full images in Supplementary Figure S9.


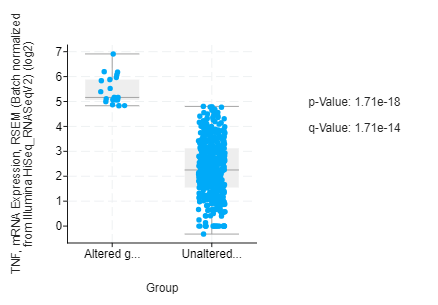


**Prostate Adenocarcinoma**

**A**

*TNF* mRNA Expression

(Log2)

*p* = 1.67E-18

**1**

**2**

**3**

**0**

**4**

**5**

**6**

**7**

Log2 Ratio = 3.18

Unaltered group

(n = 472)

Altered group

(n =20)


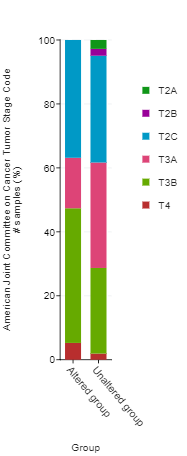


T2A

T2B

T2C

T3A

T3B

T4

N0

N1

**36.8**

**63.2**

**17.7**

**82.3**

**36.8**

**15.8**

**42.1**

**5.3**

**33**

**26.6**

**1.9**

**33.5**

**2.1**

**2.8**

**20**

**40**

**60**

**0**

**80**

**100**

**20**

**40**

**60**

**0**

**80**

**100**

Neoplasm Disease Lymph Node Stage

Sample numbers (%)

Tumor Stage

Sample numbers (%)

Tumor Stage

Altered group (n =19)

Unaltered group (n = 400)

Altered group (n = 19)

Unaltered group (n = 466)

**B**

**C**

*p* = 0.0739

*p* = 0.384

**Prostate Adenocarcinoma**

**Prostate Adenocarcinoma**

Stage

**Figure S2.** Clinical data analysis between high *TNF* mRNA (Altered) group and unaltered group in patients with prostate adenocarcinoma. (A) *TNF* mRNA expression. (B) Lymph node metastasis analysis. (C) Tumor stage analysis. Data resource: TCGA, PanCancer Atlas. Statistical test: Chi-squared test.

**C**

**D**


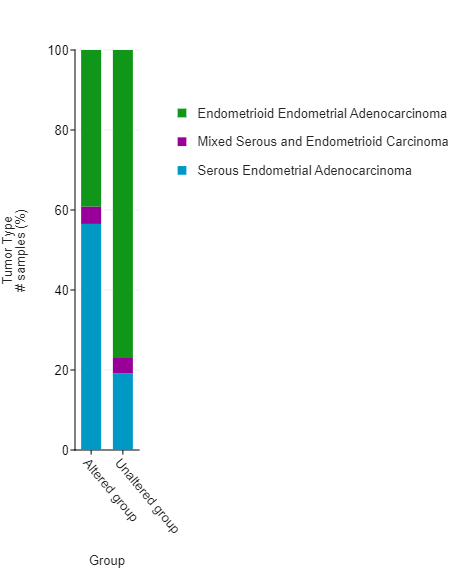


*p* = 7.873E-5

Tumor Type Sample numbers (%)

**20**

**40**

**60**

**0**

**80**

**100**

Endometrioid Endometrial

Adenocarcinoma

Mixed Serous and Endometrioid

Carcinoma

Serous Endometrial

Adenocarcinoma

Advanced Stage

Altered group (n = 23)

Unaltered group (n = 500)

**56.5**

**39.1**

**3.8**

**4.4**

**77**

**19.2**

**Uterine Corpus Endometrial Carcinoma**


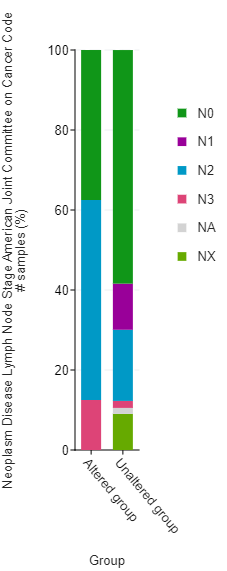


**20**

**40**

**60**

**0**

**80**

**100**

Neoplasm Disease Lymph Node Stage

Sample numbers (%)

Altered group (n = 8)

Unaltered group (n = 399)

*p* = 0.0256

N0

N1

N2

N3

NA

Stage

NX

**37.5**

**50**

**12.5**

**58.4**

**17.8**

**11.5**

**9**

**1.5**

**1.8**

**Bladder Urothelial Carcinoma**


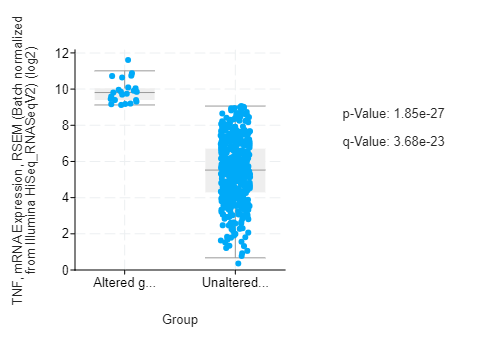


Altered group

(n =23)

Unaltered group

(n = 500)

*TNF* mRNA Expression

(Log2)

**2**

**4**

**6**

**0**

**8**

**10**

**12**

**Uterine Corpus Endometrial Carcinoma**

*p* = 1.61E-27

Log2 Ratio

= 4.41

**A**


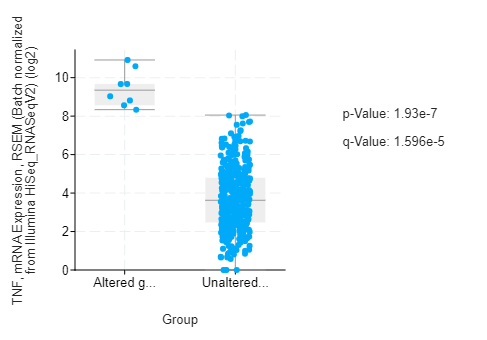


**Bladder Urothelial Carcinoma**

*TNF* mRNA Expression

(Log2)

**2**

**4**

**6**

**0**

**8**

**10**

Altered group

(n =8)

*p* = 1.93E-7

Log2 Ratio

= 5.71

**B**

Unaltered group

(n = 399)

**Figure S3.** Clinical data analysis between high *TNF* mRNA (Altered) group and unaltered group in patients with the different types of tumors. (A) *TNF* mRNA expression in uterine corpus endometrial carcinoma. (B) *TNF* mRNA expression in bladder urothelial carcinoma. (C) Tumor type analysis in uterine corpus endometrial carcinoma. (D) Lymph node metastasis analysis in bladder urothelial carcinoma. Data resource: TCGA, PanCancer Atlas. Statistical test: Chi-squared test.


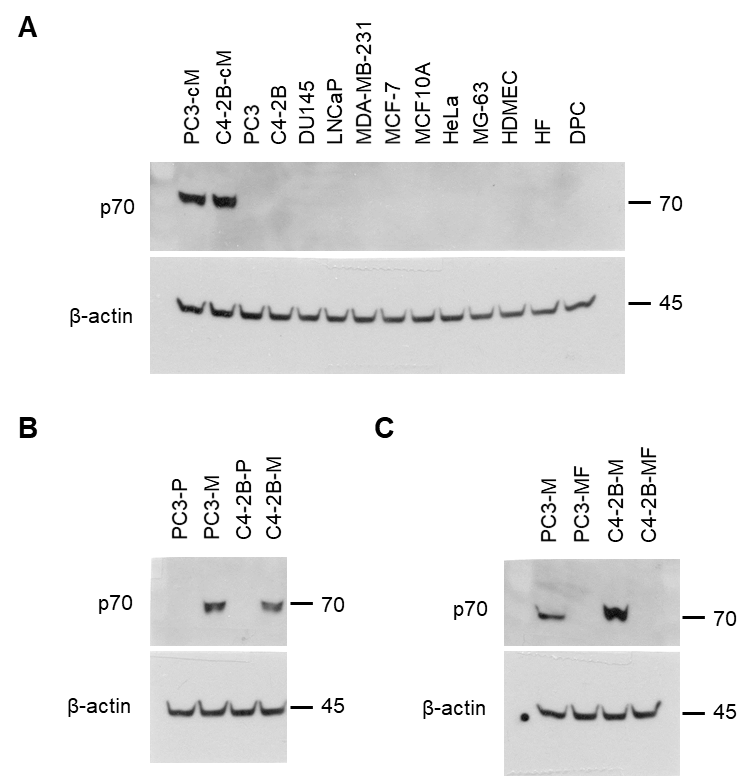


**Figure S4.** Full images of (A) Figure 1A, (B) Figure 1C, and (C) Figure 1D. *M. hyrorhinis* infection was confirmed by Western blotting using specific anti-*M.* *hyorhinis* (P70 surface antigen) antibody. β-actin was used as a loading control.


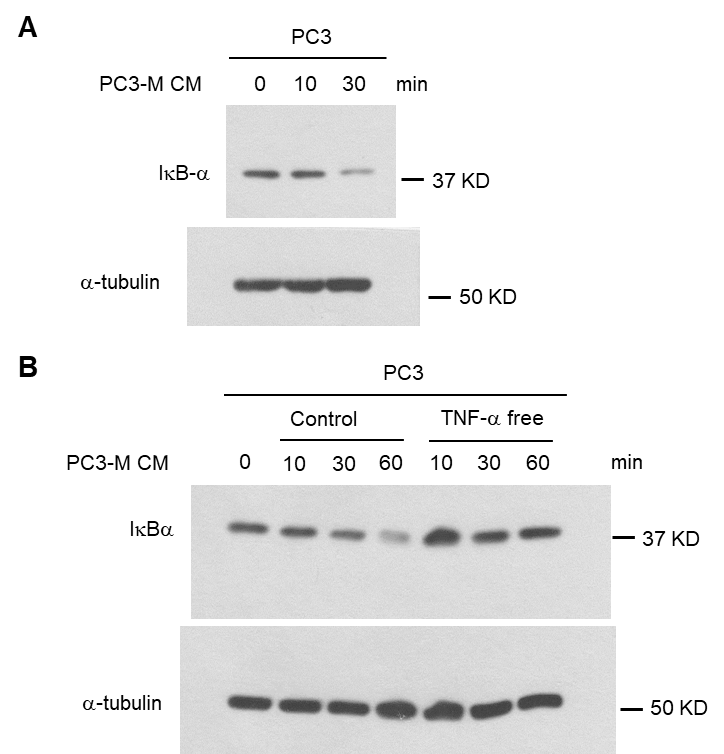


**Figure S5.** Full images of (A) Figure 4M and (B) Figure 4N. (A) Western blot analysis of IκB-α expression in PC3 cells after applying PC3-M CM for the indicated time. (B) Western blot analysis of IκB-α expression in PC3 cells after applying PC3-M CM or TNF-α free PC3-M CM for the indicated time. α-tubulin was used as a loading control.


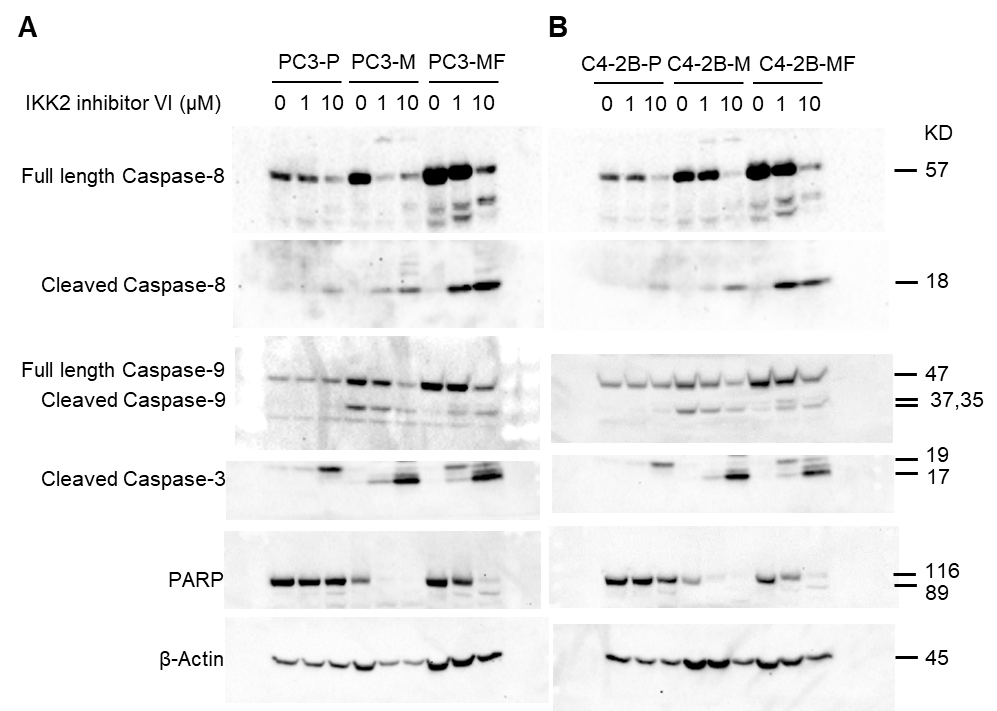


**Figure S6.** Full images of (A) Figure 5C and (B) Figure 5D. (A) Western blot analysis using Caspase-8, cleaved Caspase-8, Caspase-9, cleaved Caspase-3, and PARP antibodies in PC3-P, PC3-M, and PC3-MF cells treated with 1µM and 10 µM IKK2 inhibitor VI for 48 hours. (B) Western blot analysis using Caspase-8, cleaved Caspase-8, Caspase-9, cleaved Caspase-3, and PARP antibodies in C4-2B-P, C4-2B-M, and C4-2B-MF cells treated with 1µM and 10 µM IKK2 inhibitor VI for 48 hours. β-actin was used as a loading control.


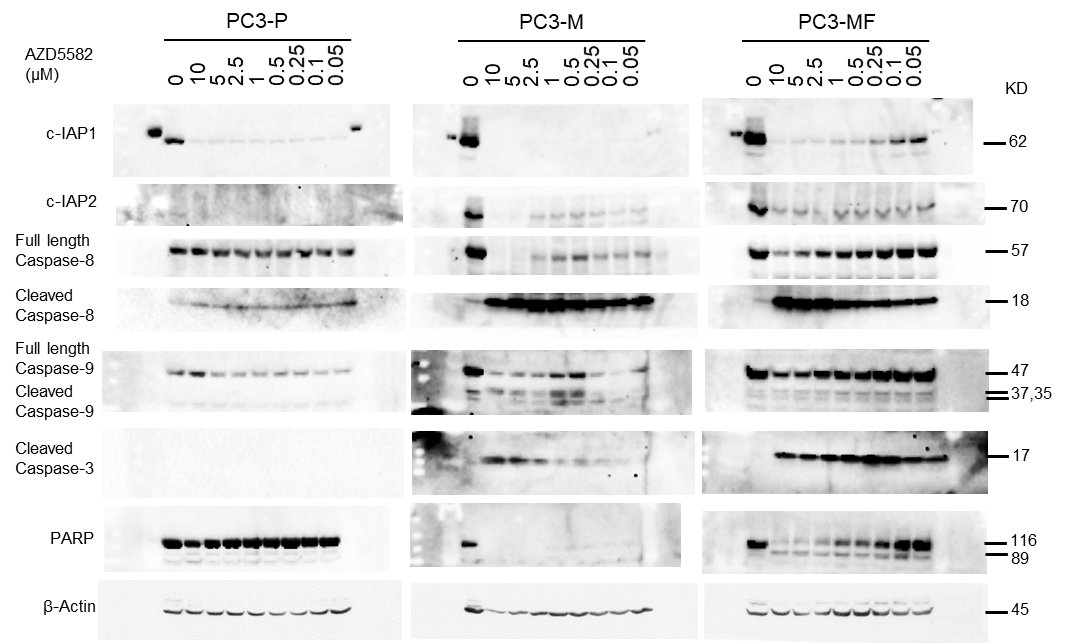


**Figure S7.** Full images of Figure 7E. Western blot analysis using c-IAP1, c-IAP2, Caspase-8, cleaved Caspase-8, Caspase-9, cleaved Caspase-3, and PARP antibodies in PC3-P, PC3-M, and PC3-MF cells treated with the indicated concentration of AZD5582 for 48 hours. β-actin was used as a loading control.


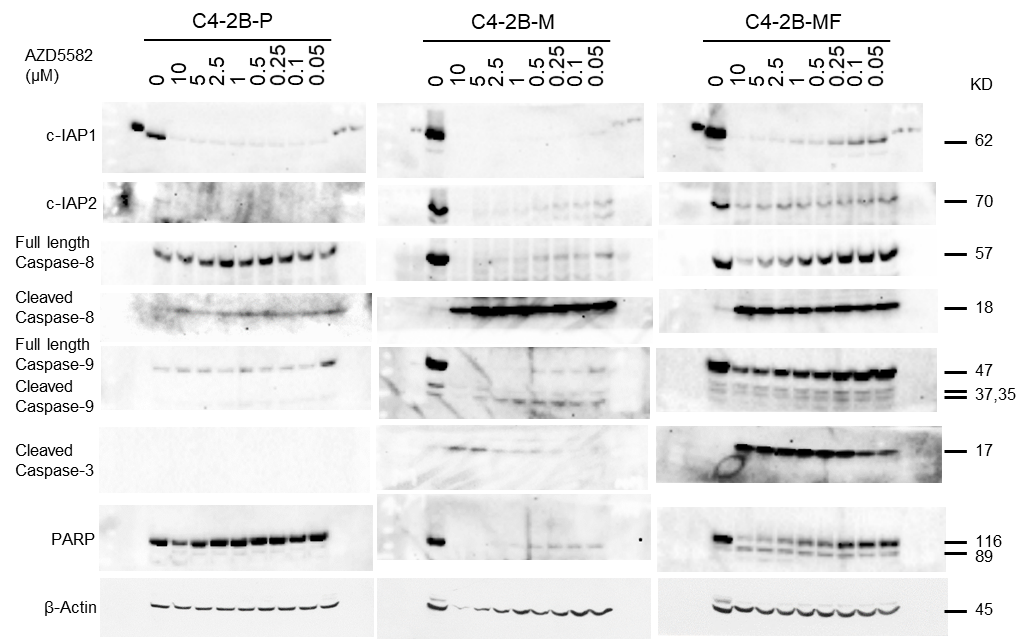


**Figure S8.** Full images of Figure 7F. Western blot analysis using c-IAP1, c-IAP2, Caspase-8, cleaved Caspase-8, Caspase-9, cleaved Caspase-3, and PARP antibodies in C4-2B-P, C4-2B-M, and C4-2B-MF cells treated with the indicated concentration of AZD5582 for 48 hours. β-actin was used as a loading control.


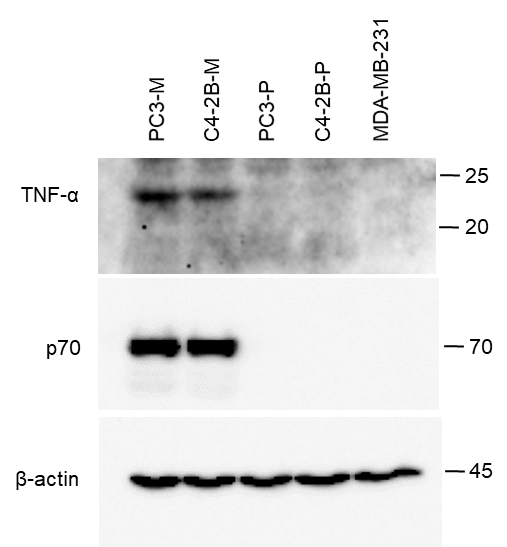


**Figure S9.** Full images of Figure S1B. Western blot analysis of TNF-α expression in *M. hyorhinis*-infected (PC3-M and C4-2B-M), parental (PC3-P and C4-2B-P), and MDA-MB-231 cells. β-actin was used as a loading control.
